# Integrated analysis of miRNAome transcriptome and degradome reveals miRNA-target modules governing floral florescence development and senescence across early- and late-flowering genotypes in tree peony

**DOI:** 10.3389/fpls.2022.1082415

**Published:** 2022-12-14

**Authors:** Lili Guo, Yuying Li, Chenjie Zhang, Zhanying Wang, John E. Carlson, Weinlun Yin, Xiuxin Zhang, Xiaogai Hou

**Affiliations:** ^1^ College of Tree Peony, Henan University of Science and Technology, Luoyang, Henan, China; ^2^ Department of Horticulture, Luoyang Academy of Agricultural and Forestry Sciences, Luoyang, Henan, China; ^3^ Department of Ecosystem Science and Management, Pennsylvania State University, University Park, PA, United States; ^4^ College of Biological Sciences and Technology, Beijing Forestry University, Beijing, China; ^5^ Center of Peony, Institute of Vegetables and Flowers, Chinese Academy of Agricultural Science, Beijing, China

**Keywords:** tree peony, miRNA-target modules, florescence, floral development, senescence

## Abstract

As a candidate national flower of China, tree peony has extremely high ornamental, medicinal and oil value. However, the short florescence and rarity of early-flowering and late-flowering varieties restrict further improvement of the economic value of tree peony. Specific miRNAs and their target genes engaged in tree peony floral florescence, development and senescence remain unknown. This report presents the integrated analysis of the miRNAome, transcriptome and degradome of tree peony petals collected from blooming, initial flowering, full blooming and decay stages in early-flowering variety *Paeonia ostii* ‘Fengdan’, an early-flowering mutant line of *Paeonia ostii* ‘Fengdan’ and late-flowering variety *Paeonia suffruticosa* ‘Lianhe’. Transcriptome analysis revealed a transcript (*‘psu.G.00014095’*) which was annotated as a xyloglucan endotransglycosylase/hydrolase precursor *XTH-25* and found to be differentially expressed across flower developmental stages in *Paeonia ostii* ‘Fengdan’ and *Paeonia suffruticosa* ‘Lianhe’. The miRNA-mRNA modules were presented significant enrichment in various pathways such as plant hormone signal transduction, indole alkaloid biosynthesis, arachidonic acid metabolism, folate biosynthesis, fatty acid elongation, and the MAPK signaling pathway. Multiple miRNA-mRNA-TF modules demonstrated the potential functions of *MYB*-related, *bHLH*, *Trihelix*, *NAC*, *GRAS* and *HD-ZIP* TF families in floral florescence, development, and senescence of tree peony. Comparative spatio-temporal expression investigation of eight floral-favored miRNA-target modules suggested that transcript ‘*psu.T.00024044*’ and microRNA *mtr-miR166g-5p* are involved in the floral florescence, development and senescence associated agronomic traits of tree peony. The results might accelerate the understanding of the potential regulation mechanism in regards to floral florescence, development and abscission, and supply guidance for tree peony breeding of varieties with later and longer florescence characteristics.

## 1 Introduction

Tree peony (*Paeonia suffruticosa* Andr.) is a perennial deciduous shrub of *Paeoniaceae.* All kinds of tree peony species are endemic to China (Wang et al., 2019). Tree peony, famous for ‘the king of flowers’, is big, colorful, fragrant, graceful and elegant, symbolizing wealth and prosperity, and is renowned as a symbol of Chinese civilization (Yang et al., 2020). The poem ‘Only peony is the true color of the country, which moves the capital when they are blossoming’, vividly describes the people’s deep love for peony flowers in the Tang Dynasty. These days, tree peonies are cultivated all over the world, and people’s enthusiasm to cultivate and plant tree peonies is still increasing (Hong et al., 2017).

Research on ornamental characters of tree peony has made continuous progress from identification and cultivation of varieties to improved cultivation technology. There are already more than 2,000 ornamental varieties of tree peony in China (Luo et al., 2021). However, the flowering time of tree peony is still quite short and convergent. Under natural conditions, it takes only 50-60 days from budding to fading; the flowering period is 3-5 days for a single flower and 10-15 days for a colony of plants. Most tree peony varieties are middle-flowering varieties, with the proportion of early-flowering and late-flowering varieties being quite few (Li et al., 2011). In addition, due to the lack of research on the genetic basis and molecular regulation mechanism of flowering in tree peony, it is difficult to improve the breeding and cultivation techniques to meet the demand for prolonging of flowering time of tree peony. These are all important factors restricting the improvement of tree peony ornamental value and the further development of international markets (Kamenetsky et al., 2003). Florescence has thus been one of the key ornamental traits that limit the improving of the economic value of tree peony, and the theoretical and technical research needed to solve this problem has become a key focus in both the public and scientific horticulture communities.

In efforts to prolong the ornamental period of tree peony, studies have been carried out from such aspects as early and late flowering hybrid breeding and growth regulator regulation. However, conventional breeding of woody perennials in tree peony is time-consuming and labor-intensive making it difficult to quickly meet the market demand for new varieties. Although high-density genetic maps and QTL identification are now being reported, the low breeding efficiency greatly currently remains a limiting factor in the selection and breeding process of tree peony varieties (Cai et al., 2015; Li et al., 2019; Zhang et al., 2019). It should thus be of great value to construct the transcriptional regulatory network for blooming, to reveal the regulation basis of early and late blooming, and explore the application of genetic regulation factors for accelerating the breeding of tree peony varieties with extra-early, late, and long-lasting flowers.

MicroRNAs (miRNA) are short (21-24 nucleotide) RNAs originated from noncoding RNAs root in the expression of miRNA genes (MIR genes) (Basso et al., 2019). MiRNAs have become crucial modulator of gene expression, primarily by means of the cleavage/inhibit of target genes translation during or after transcription (Xie et al., 2020). MiRNAs regulate almost all the crucial biological processes of the plants’ life cycle, such as growth and development (Li et al., 2019), flowering (Spanudakis and Jackson, 2014), ripening (Guo et al., 2018), postharvest senescence (Pei et al., 2013; Chen et al., 2020), and plant-environment interactions (Basso et al., 2019). MiRNAs can rapidly reprogram the expression patterns of downstream genes that strictly regulate agronomic trait, for instance, florescence (Wang et al., 2009). Previous studies have emphasized the significance of miRNAs involved in floral transition and flowering regulation (Waheed and Zeng, 2020). Studies of *miR156*, *miR172*, *miR390*, *miR159*, *miR169*, and *miR399* have shown that they are key factors affecting flowering time (Waheed and Zeng, 2020).

Integrated analysis of the miRNAome, transcriptome, and degradome analyses can enhance the understanding of the genome wide co-expression patterns of miRNA-mRNA pairs and links the biological interactions of miRNA-target modules (Liu et al., 2020a; Wang et al., 2021). Integrated miRNA, transcriptome, and degradome-seq analysis of miRNA-mRNA involved in flowering of pepper (Shu et al., 2021), floral development and abscission of yellow lupine (Glazinska et al., 2019), female sterility of pomegranate (Chen et al., 2020), male sterility of rice (Sun et al., 2021), flower development across capsicum species (Lopez-Ortiz et al., 2021), stamen development in moso bamboo (Cheng et al., 2019), flowering induction in *Lilium*×*formolongi* (Zhang et al., 2021), floral transition in *Magnolia*×*soulangeana* ‘Changchun’ (Sun et al., 2021) has provided evidence demonstrated of regulatory pathways and gene networks of miRNAs and their targets associated with flowering. Moreover, studies have shown that *miR319*-*TCP*, *miR156*-*SPL*, *miR159*-*MYB*, *miR172*-*AP2* and *miR399*-*PHO2* nodes play important roles in floral transition (Waheed and Zeng, 2020).

Prediction of miRNAs in tree peony have been reported, including miRNAs in response to copper stress (Jin et al., 2015), involved in bud dormancy release (Zhang et al., 2018), seed fatty acid synthesis (Yin et al., 2018), flower spot formation (Zhao et al., 2019), petal variegation (Shi et al., 2019), flower development (Han et al., 2020), and brassinolide treatment on flowering (Zhang et al., 2022). In herbaceous peony, miRNAs involved in response to stress from high temperature (Hao et al., 2017) and *Botrytis cinerea* infection (Zhao et al., 2015), as well as lateral branch formation (Liu et al., 2020) have been predicted. In addition, *miR156e-3p* of herbaceous peony has been proved to enhance anthocyanin accumulation in lateral branches of transgenic *Arabidopsis thaliana* (Zhao et al., 2017). However, currently, miRNAome analysis during reproductive growth, has not yet been applied to the elucidation of miRNA-mRNA module regulatory networks specific to the trait of flowering among tree peony varieties with contrasting flowering times.

In this study, we identified and determined the critical miRNA and their MIR genes using miRNAome analysis combined with transcriptome, degradome and qRT-PCR verification during flower development stages in three tree peony varieties with different flowering times. This research might enlighten the composition of post-transcriptional networks in tree peony floral florescence, development and abscission, and facilitate innovations for breeding programs aiming to prolong the flowering period.

## 2 Materials and methods

### 2.1 Materials preparation

Early-flowering variety *Paeonia ostii* ‘Fengdan’ (FD), an early-flowering mutant line of *Paeonia ostii* ‘Fengdan’ (MU), and late-flowering variety *Paeonia suffruticosa* ‘Lianhe’ (LH) were selected as the experiment materials. FD, MU, and LH used in this study were 13-years-old plants with single and white flowers. Fresh petals were collected at 9:00-10:00 am on different days at blooming stage (BS), initial flowering stage (IF), full bloom stage (FB), and decay stage (DE), respectively. Abbreviations of sample and library names were presented in **Table 1**. Three biological replicates are different flowers on different stems of the same plant at each developmental stage for each variety respectively. For each flower, all the petals were sampled and pooled prior to freezing by liquid nitrogen. The sampled petals were stored in the freezer (-80°C) for RNA extraction.

**Table 1:** Summary of the abbreviations used for sample names and library names.

Type of Terms	Abbreviations	Description
*Genotype*	*MU*	*Mutant of Paeonia ostii ‘Fengdan’, opening earlier than Paeonia ostii ‘Fengdan’*
	*FD*	*Paeonia ostii ‘Fengdan’, early flowering cultivar*
	*LH*	*Paeonia suffruticosa ‘Lianhe’, late flowering cultivar*
*Developmental stage*	*CE*	*Color Exposure Stage*
	*BS*	*Blooming Stage*
	*IF*	*Initial Flowering Stage*
	*HO*	*Half Opening Stage*
	*FB*	*Full Blooming Stage*
	*ID*	*Initial Decay Stage*
	*DE*	*Decay Stage*

### 2.2 RNA isolation, library construction and sequencing

In total, 36 libraries were prepared for miRNAome and transcriptome analysis separately (**Table S1**). Approximately 200 mg petals were used for total RNA extraction. Total RNA integrity was initially assessed by denaturing agarose gel electrophoresis, then confirmed by Bioanalyzer 2100 (Agilent, CA, USA). Total RNA amount and purity quantification were performed on NanoDrop ND-1000 (NanoDrop, Wilmington, DE, USA). Total RNA with a concentration>100 ng/μL, RNA integrity number >7.0, OD260/280>1.8 and amount>50 μg were used for library construction. Petals from four flower development stages of each variety were mixed prior to the degradome library construction. That is, a total of 3 degradome libraries were constructed for degradome sequencing. Kits and reagents used for RNA isolation, purification, quantification, and libraries construction are listed in **Table S2**. Transcriptome sequencing was performed by the 2×150 bp paired-end sequencing (PE150) on Illumina Novaseq™ 6000. miRNAome and degradome sequencing were performed by the 1×50 bp single-end sequencing on Illumina Hiseq2500. Libraries construction and sequencing were performed at LC-BIO (Hangzhou, China) according to the vendor’s recommended protocol.

### 2.3 Data processing of miRNAome, transcriptome, and degradome

#### 2.3.1 Data processing of miRNAome

Raw data filtering was processed using ACGT101-miR (LC Sciences, Houston, Texas, USA). Sequences with a length of 18-26 nt were mapped to the genome of tree peony (https://ftp.cngb.org/pub/CNSA/data1/CNP0000281/CNS0044072/CNA0002540/) (Lv et al., 2020) and miRBase 22.0 (http://www.mirbase.org/). Sequences mapped to miRBase 22.0 were characterized as known miRNA. Sequences unmapped to miRBase 22.0 and matched to tree peony genome were identified as candidate novel miRNAs. Secondary structure prediction of miRNA was performed by RNAfold (http://rna.tbi.univie.ac.at/cgi-bin/RNAWebSuite/RNAfold.cgi). Sequences possess stem-loop structure and satisfy the requirement of miRNA prediction (Axtell and Meyers, 2018) were considered as real miRNAs of tree peony. Differentially expressed miRNA (DEM) analysis based on normalized (Li et al., 2016) deep-sequencing counts was performed by ANOVA with the criterion of *P ≤* 0.05. DEM target genes prediction was performed by PsRobot 1.2 to characterize the miRNA binding sites.

#### 2.3.2 Data processing of transcriptome

Raw data filtering was conducted by FASTP (https://github.com/OpenGene/fastp) to remove reads containing adaptor contamination, low quality bases and undetermined bases. FastQC (http://www.bioinformatics.babraham.ac.uk/projects/fastqc/, 0.11.9) and FASTP was used for sequences quality verification. HISAT2 (https://ccb.jhu.edu/software/hisat2) was used for the reads mapping to the reference genome of tree peony (Lv et al., 2020). Reads were assembled by StringTie (http://ccb.jhu.edu/software/stringtie/ ) (Version: stringtie-1.3.4d.Linux_x86_64). Data merging was conducted using gffcompare http://ccb.jhu.edu/software/stringtie/gffcompare.shtml). Transcripts’ expression levels were estimated by StringTie according to FPKM method (FPKM=[total_exon_fragments/mapped_reads(millions)×exon_length(kB)]). Identification of DEGs with fold change>2 or <0.5 and *P* value<0.05 were performed using edgeR (https://bioconductor.org/packages/release/bioc/html/edgeR.html). GO and KEGG pathway investigation were performed by DAVID (https://david.ncifcrf.gov/). TFs were investigated by iTALK (v1.2) software596. WGCNA was performed according to Langfelder and Horvath (2008).

#### 2.3.3 Data processing of degradome

Degradome data processing was performed by program ACGT10-DEG (LC Sciences, Houston, Texas, USA) using software package CleaveLand4 according the following command: degradome and transcriptome data alignment and generate a degradome density file, miRNAs and transcriptome alignment to parse miRNA-mRNA potential target site, cross-referencing to the degradome data to demonstrate the slicing site.

### 2.4 miRNAs and targets expression assay

In total, eight miRNA-target pairs associated with floral florescence, development and senescence were randomly selected for quantitative real-time PCR (qRT-PCR) analysis. Fresh petals of FD, MU and LH were collected at developmental stages (BS, IF, FB, DE) respectively. For each developmental stage, petals from three different flowers on different stems of the same plant were sampled individually. Total RNA extraction, miRNA extraction, cDNA synthesis for mRNA and miRNA were performed according to the instructions of manufacturers. Kits information for total RNA/miRNA extraction and cDNA synthesis are shown in **Table S2**. The *EF1-α* and *U6* were used as the reference for mRNA and tailing reaction miRNA analysis separately. Primer sequences for qRT-PCR assay are listed in **Table S3**. qRT-PCR analysis for miRNA and targets were both performed on a BIORAD CFX96 machine. Three technical replicates per reaction were conducted in the qRT-PCR analyses to ensure statistical validity. The relative quantity was calculated on the basis of 2^−ΔΔCT^ method (Livak and Schmittgen, 2001).

## 3 Results

### 3.1 Morphological comparison of flowering time in three tree peony varieties

**Figure 1:**
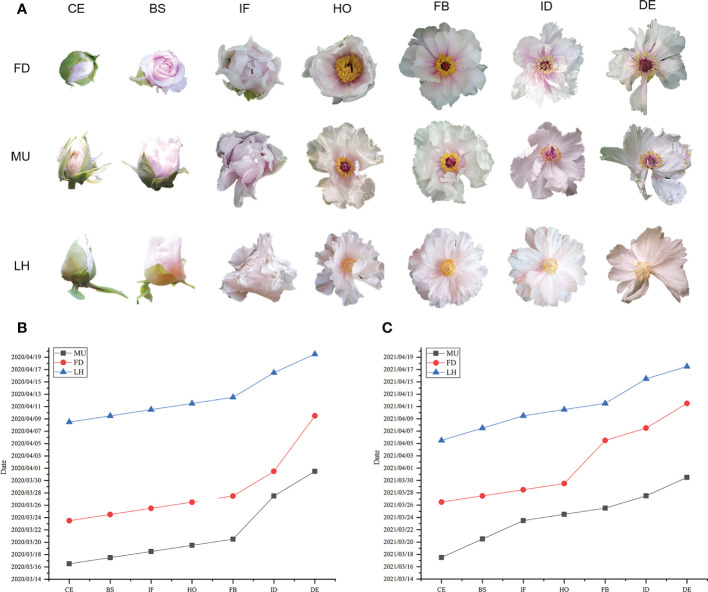
Phenotype and flowering time investigation of FD, MU, and LH. **(A)** Flower phenotype at different developmental stages of FD, MU, and LH. **(B)** The date when 80% flowers reached different development stages in FD, MU, and LH investigated in 2020. **(C)** The date when 80% flowers reached different development stages in FD, MU, and LH investigated in 2021.

The date when 80% of flowers reached color exposure (CE), blooming stage (BS), initial flowering (IF), half opening (HO), full blooming (FB), initial decay (ID), and decay (DE) stages were investigated in FD, MU, and LH in 2020 and 2021 respectively (**Figure 1A**). The flower duration time (date from CE to DE) of 80% flowers of FD, MU, LH were also investigated. FD is an early flower variety, while LH is a late flowering variety. MU was a mutant of FD, which blossomed seven days earlier than FD in 2020, and nine days earlier than FD in 2021. Flowering time of FD was 16 days earlier than LH in 2020, and 10 days earlier than LH in 2021. Flowering time of MU was 23 and 19 days earlier than LH in 2020 and 2021, respectively. In addition, floral florescence per plant was 16-17 days for FD, 13-15 days for MU, and 11-12 days for LH, which demonstrated that FD possesses the longest blooming time (**Figures 1B, C**).

### 3.2 MiRNAome analysis

#### 3.2.1 Expression miRNAs of tree peony revealed by miRNAome

Petal samples were collected at BS, IF, FB, and DE stages from three replicates of each from the three varieties, respectively. An overview of miRNAome sequencing data is presented in **Table S4**. Over 650.34 million raw reads were produced, therein to, 123.55 million filtered miRNA reads were acquired. A total of 252, 297, and 263 miRNAs were determined to be expressed in common across the four developmental stages (BS, IF, FB, and DE) in FD, MU, and LH, respectively (**Figures S1A–C**). There are altogether 290, 311, 252, and 226 miRNAs expressed in common across the varieties (FD, MU, and LH) at developmental stages BS, IF, FB, and DE, respectively (**Figures S1E–H**). Finally, a total of 164 miRNA were expressed in common across all four developmental stages and three varieties (**Figures S1D, I**).

#### 3.2.2 Known and predicted miRNAs of tree peony revealed by sequencing the miRNAome

In total, 2,444 pre-miRNA and 2,617 unique miRNAs were identified by miRNAome analysis (**Table 2**). The miRNAs were classified into known and predicted groups (**Table S5**). Four types of known miRNAs were included in group1 (gp1), group2a (gp2a), group1 (gp2b), and group1 (gp3), while only predicted miRNAs were included in group4 (gp4). In ‘gp1’ were placed the reads that mapped to specific miRNAs/pre-miRNAs in miRbase for which the pre-miRNAs mapped to the tree peony genome and to the ESTs. In ‘gp2a’ were placed reads that mapped to selected miRNAs/pre-miRNAs in miRbase, but for which the pre-miRNAs did not map to the tree peony genome. However, the reads (the miRNAs of the pre-miRNAs) in this group did map to the tree peony genome. Also, the extended sequences from the corresponding loci in the tree peony genome which could form hairpins. In ‘gp2b’ were placed reads that mapped to miRNAs/pre-miRNAs of selected species in miRbase. The pre-miRNAs did not map to the tree peony genome, however, the reads (the miRNAs from the pre-miRNAs) did map to the tree peony genome. Also, the extended sequences at the genome loci could not form hairpins in this case. In ‘gp3’ were placed reads that mapped to the selected miRNAs/pre-miRNAs in miRbase but for which the pre-miRNAs do not map to the tree peony genome, and also the reads did not map to the tree peony genome. In ‘gp4’ were placed reads that did not map to the selected pre-miRNAs in miRbase. However, these reads did map to the tree peony genome and the extended sequences from the genome loci which could form hairpins. In summary, for pre-miRNA, a total of 735 known miRNAs including gp1 (10), gp2a (75), gp2b (611), gp3 (39) were identified, and a total of 1,709 predicted miRNAs which only include gp4 were characterized. For unique miRNA, a total of 796 known miRNAs including gp1 (15), gp2a (106), gp2b (632), gp3 (43) were defined, and a total of 1,812 predicted miRNAs which only include gp4 were detected (**Table 2**).

**Table 2:** Summary of known and predicted miRNA.

	groups	pre-miRNA	total	unique miRNA	total
known miRNA	group1	10	735	15	796
	group2a	75		106	
	group2b	611		632	
	group3	39		43	
predicted miRNA	group4	1709	1709	1812	1821
Total		2444		2617	

#### 3.2.3 Differentially expressed miRNAs among stages in floral florescence development

To identify DEMs engaged in floral florescence and senescence, significantly differential expressed miRNAs (*P*<0.01, *P*<0.05, *P*<0.1) were analyzed across four developmental stages and three varieties. In total, 146, 313, and 201 miRNAs showed signifcant differential expression (*P*<0.05) across four developmental stages in FD, MU, and LH, respectively. A total of 253, 227, 285, 270 miRNAs revealed signifcant differential expression (*P*<0.05) across three varieties (FD, MU, LH) at stage of BS, IF, FB, and DE separately. The numbers of differentially expressed up- and down-regulated miRNAs in different groups are shown in **Figure S2**. Up-regulated DEMs refer to the miRNAs having signifcantly higher expression, while down-regulated DEMs refer to the miRNAs which present prominently lower expression.

**Figure 2:**
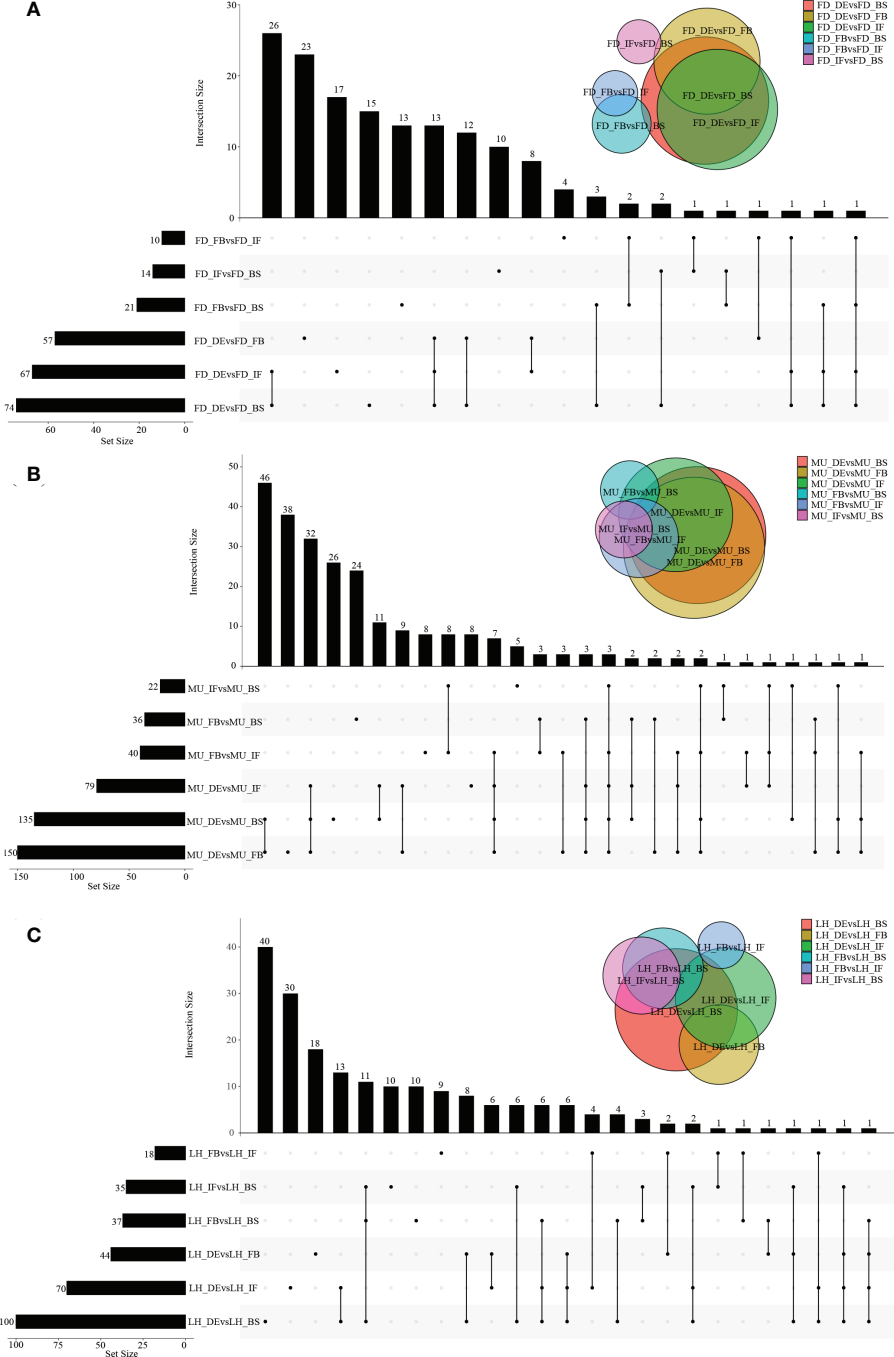
The distribution of DEMs across flower developmental stages in FD, MU, and LH. **(A)** The distribution of DEMs across developmental stages (BS, IF, FB, DE) in FD libraries. **(B)** The distribution of DEMs across developmental stages (BS, IF, FB, DE) in MU libraries. **(C)** The distribution of DEMs across developmental stages (BS, IF, FB, DE) in LH libraries.

**Figure 3:**
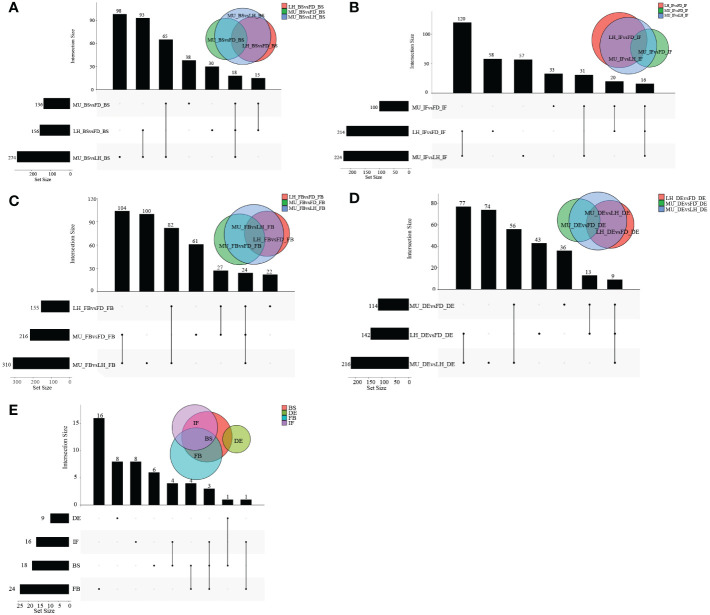
The distribution of DEMs across varieties at BS, IF, FB, and DE stages. **(A)** The distribution of DEMs across tree peony varieties (FD, MU and LH) at BS libraries. **(B)** The distribution of DEMs across tree peony varieties (FD, MU and LH) at IF libraries. **(C)** The distribution of DEMs across tree peony varieties (FD, MU and LH) at FB libraries. **(D)** The distribution of DEMs across tree peony varieties (FD, MU and LH) at DE libraries. **(E)** The distribution of intersection DEMs across tree peony varieties (FD, MU and LH) and across four flower developmental stages (BS, IF, FB, DE).

A wide variety of miRNAs showed differential expression specific to genotype and developmental stage. However, no DEMs were found to be co-expressed across the four developmental stages even in a single variety, which meant the analysis of co-expressed DEMs between varieties based on intersection of DEMs across developmental stages could not be performed. In fact, higher numbers of differentially expressed miRNAs were presented in DEvsBS, DEvsFB, and DEvsIF when comparing DEMs across developmental stages irrespective of variety. MU constantly had a higher number of DEMs commonly expressed when compared with FD and LH (**Figures 2A–C**). When comparing across varieties (FD, MU and LH) at specific developmental stages, 18, 16, 24 and 9 intersecting DEMs were obtained at developmental stages BS, IF, FB and DE respectively (**Figures 3A–D**). Meanwhile, there were more DEMs exclusively expressed in the FB stage than BS, IF and DE stages in all three of the varieties (**Figures 3A–D**). Interestingly however, three DEMs were identified that simultaneously expressed in the BS, IF, and FB stages. Finally, the expression of 6, 8, 16, and 8 stage-specific DEMs, at BS, IF, FB and DE stages, respectively, were not affected by variety (**Figure 3E**).

### 3.3 Transcriptome analysis

#### 3.3.1 Expression genes revealed by transcriptome sequencing

Overview of raw reads, valid reads, Q30 and GC content data are shown in **Table S6**. Over 227 million valid reads, after filtering of 245 million raw reads, were obtained. In total, 500,378 contigs, 35,687 genes (G), 35,687 unique Transcripts (T) were identified separately. Of these, 29,271 GO annotated genes and 10,556 KEGG annotated genes were obtained separately. A total of 15,099, 14,906, and 14,544 intersecting annotated genes were detected across developmental stages (BS, IF, FB, and DE) in FD, MU, and LH varieties respectively (**Figures S3A–C**). A total of 14,672, 14,844, 14,841, and 14,779 intersecting annotated genes were detected across all varieties (FD, MU, and LH) at all developmental stages BS, IF, FB, and DE respectively (**Figures S4A–D**). Finally, a total of 13,203 intersecting annotated genes were detected across four developmental stages and three varieties (**Figure S3D**, **Figure S4E**).

#### 3.3.2 Differentially expressed genes identified in floral florescence development stages

**Figure 4:**
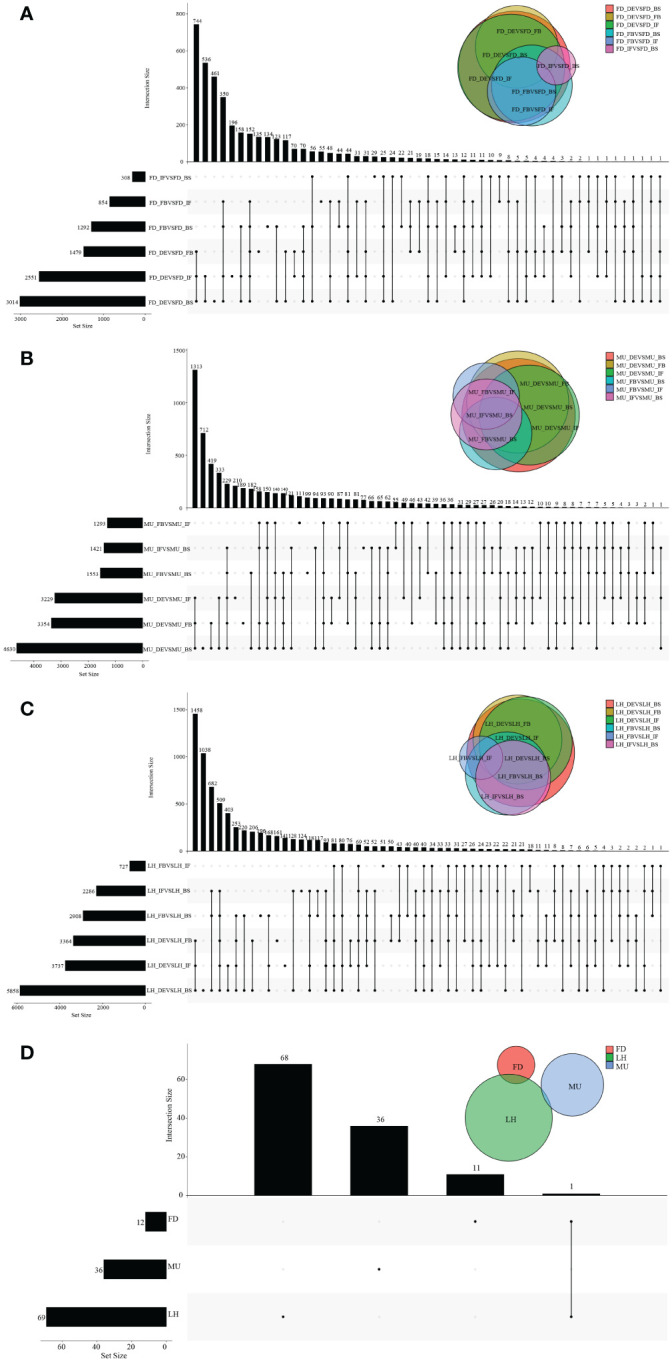
The distribution of DEGs across flower developmental stages in FD, MU, and LH. **(A)** The distribution of DEGs across flower developmental stages (BS, IF, FB, DE) in FD libraries. **(B)** The distribution of DEGs across flower developmental stages (BS, IF, FB, DE) in MU libraries. **(C)** The distribution of DEGs across flower developmental stages (BS, IF, FB, DE) in LH libraries. **(D)** The distribution of intersection DEGs across four flower developmental stages (BS, IF, FB, DE) and across the three tree peony varieties (LH, MU and LH).

**Figure 5:**
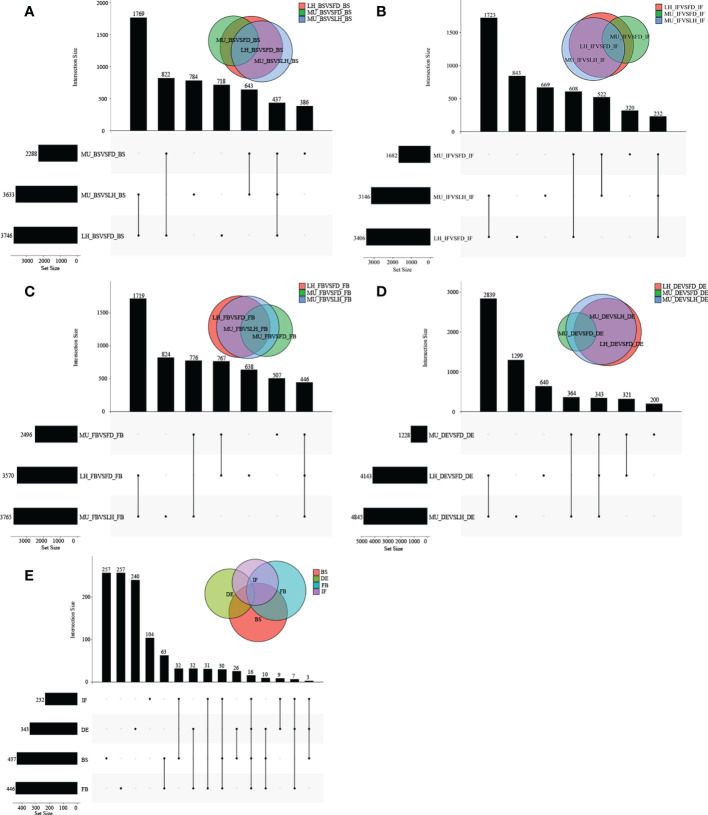
The distribution of DEGs across varieties at BS, IF, FB, and DE stages. **(A)** The distribution of DEGs across tree peony varieties (LH, MU and LH) at BS libraries. **(B)** The distribution of DEGs across tree peony varieties (LH, MU and LH) at IF libraries. **(C)** The distribution of DEGs across tree peony varieties (LH, MU and LH) at FB libraries. **(D)** The distribution of DEGs across tree peony varieties (LH, MU and LH) at DE libraries. **(E)** The distribution of intersection DEGs across the three tree peony varieties (LH, MU and LH) and across the four flower developmental stages (BS, IF, FB, DE).

To identify differentially expressed genes (DEGs) with floral florescence patterns, gene expression was compared across developmental stages in each of the varieties (**Figures 4A–C**). Numbers of differentially expressed DEGs including up-regulated and down-regulated DEGs in different groups are shown in **Figure S5**. A total of 12, 36, and 69 genes showed significant differential expression among flower developmental stages (BS, IF, FB, DE) in FD, MU and LH varieties, respectively (**Figure 4D**). Interestingly, after determining the intersection of differentially expressed genes in the three varieties, we identified only one co-expressed DEG (psu.G.00014095), in varieties FD and LH, that was expressed across all four development stages, which indicates that expression of DEG psu.G.00014095 was dependent both on developmental stage and genotype. The function of this gene was annotated as xyloglucan endotransglycosylase/hydrolase precursor *XTH-25*, which may regulate the floral florescence, development and senescence of tree peony. In addition, genes differentially expressed DEGs at different developmental stages across all varieties were identified to discover genes that may regulate flowering (**Figures 5A–D**). In total, 437, 232, 446, and 343 co-expressed genes showed significant differential expression among stages BS, IF, FB, and DE, respectively, across varieties FD, MU and LH (**Figure 5E**). Examining intersections of the DEGs further revealed 16 DEGs that simultaneously expressed at the four developmental stages and in the three varieties (**Figure 5E**). Besides, 257, 104, 257, and 240 specific DEGs showed significant differential expression across in stages in FB, IF, FB, and DE, respectively, in all the three varieties (**Figure 5E**).

#### 3.3.3 Gene ontology and kyoto encyclopedia of genes and genomes analysis

Gene Ontology (GO) and Kyoto Encyclopedia of Genes and Genomes (KEGG) based functional enrichment analysis of DEGs were conducted to uncover the biological roles in governing floral florescence, development and abscission across early- and late-flowering genotypes in tree peony.

Some GO terms (chloroplast, chloroplast envelope, plasmodesma, and molecular function) for DEGs were common across both varieties and developmental stages, while some were specific to either the variety (e.g., defense response) or to developmental stages (flavonoid biosynthetic process, flavonoid glucuronidation, quercetin 7-o-glucosyltransferase activity, quercetin 3-o-glucosyltransferase activity, chloroplast stroma, kinase activity, response to cold, chloroplast thylakoid membrane, thylakoid, chloroplast thylakoid). It was worth noting that GO terms like abscisic acid-activated signaling pathway and protein serine/threonine kinase activity processes were significantly enriched across developmental stages in FD and MU which were early-flowering genotype, while biological process, plant type cell wall, vacuole, hydrolase activity, hydrolyzing o-glucosyl compounds, golgi apparatus, transferase activity, transferring glycosyl groups, fatty acid biosynthetic process, chloroplast inner membrane processes were only enriched across developmental stages in LH which was late-flowering genotype. The GO terms for response to abscisic acid, wounding, chitin, and protein phosphorylation were only enriched in MU, which was an early flowering mutant genotype of FD (**Table 3**).

**Table 3:** GO enrichment analysis of DEGs across genotypes and developmental stages in tree peony.

GO term	Sample
Chloroplast	A, B, C, D, E, F, G
Chloroplast envelope	A, B, C, D, E, F, G
Plasmodesma	A, B, C, D, E, F, G
Molecular function	A, B, C, D, E, F, G
Defense response	A, B, C, E, F
Flavonoid biosynthetic process	A, B, D, E, F, G
Flavonoid glucuronidation	A, B, D, E, F, G
Quercetin 7-o-glucosyltransferase activity	A, B, D, E, F, G
Quercetin 3-o-glucosyltransferase activity	A, B, D, E, F, G
Chloroplast stroma	B, C, D, E, F, G
Kinase activity	B, C, D, E, F, G
Response to cold	B, C, D, E, F, G
Chloroplast thylakoid membrane	D, E, F, G
Thylakoid	D, E, F, G
Chloroplast thylakoid	D, E, F, G
Abscisic acid-activated signaling pathway	A, B, G
Protein serine/threonine kinase activity	A, B
Extracellular region	A
Plasma membrane	A
Regulation of organ growth	A
Monooxygenase activity	A, G
Protein phosphatase inhibitor activity	A, G
Abscisic acid binding	A, G
Protein serine/threonine phosphatase activity	A, F, G
Response to wounding	B, E, F
Response to abscisic acid	B
Protein phosphorylation	B
Response to chitin	B
Biological process	C, D, F, G
Plant type cell wall	C, D
Vacuole	C, E
Hydrolase activity, hydrolyzing O-glucosyl compounds	C
Golgi apparatus	C
Transferase activity, transferring glycosyl groups	C
Fatty acid biosynthetic process	C
Chloroplast inner membrane	C
Oxidation-reduction process	A, C, F
Anchored component of plasma membrane	A, C
Apoplast	B, C, D, F
Cell wall	B, C
Response to water deprivation	D, E
Response to heat	D
Response to salt stress	D
Response to light stimulus	E
Plastoglobule	E

A: Samples collected from FD across four developmental stages. B: Samples collected from MU across four developmental stages. C: Samples collected from LH across four developmental stages. D: Samples collected from BS stage across three genotypes. E: Samples collected from IF stage across three genotypes. F: Samples collected from FB stage across three genotypes. G: Samples collected from DE stage across three genotypes.

KEGG enrichment analysis of DEGs revealed that plant hormone signal transduction, starch and sucrose metabolism, MAPK signaling pathway-plant, phenylpropanoid biosynthesis, and carotenoid biosynthesis pathway were common across developmental stages in both early- and late-flowering varieties, which suggests a possible role in the floral florescence, development and abscission in tree peony. Here, it was interesting that KEGG pathways like cyanoamino acid metabolism, galactose metabolism, other glycan degradation, fatty acid elongation, and amino sugar and nucleotide sugar metabolism were specific to varieties, while some other pathways were specific to developmental stages (for example, porphyrin and chlorophyll metabolism and fructose and mannose metabolism). Thus, these KEGG pathways might possibly have roles in variety-specific or developmental stage-specific responses. It is worth mentioning that pathways for stilbenoid, diarylheptanoid and gingerol biosynthesis, glycosphingolipid biosynthesis-ganglio series, arachidonic acid metabolism, terpenoid backbone biosynthesis, and zeatin biosynthesis were common to the early-flowering varieties FD and MU, while fructose and mannose metabolism, sphingolipid metabolism, fatty acid biosynthesis, inositol phosphate metabolism, pyruvate metabolism, phosphatidylinositol signaling system were specific to the late-flowering genotype LH, which may contribute to the late-flowering phenotype (**Table 4**).

**Table 4:** KEGG enrichment analysis of DEGs across genotypes and developmental stages in tree peony.

Pathway name	Sample
Starch and sucrose metabolism	A, B, C, D, E, F, G
Carotenoid biosynthesis	A, B, C, D, E, F, G
MAPK signaling pathway-plant	A, B, C, D, E, F, G
Phenylpropanoid biosynthesis	A, B, C, D, E, F, G
Plant hormone signal transduction	A, B, C, D, E, F, G
Cyanoamino acid metabolism	A, B, C, E, F, G
Galactose metabolism	A, B, C, D, E, F
Other glycan degradation	A, B, C, D, E, F
Fatty acid elongation	A, B, C, D, E
Amino sugar and nucleotide sugar metabolism	A, B, C
Porphyrin and chlorophyll metabolism	A, B, D, E, F, G
Fructose and mannose metabolism	C, D, E, F, G
Stilbenoid, diarylheptanoid and gingerol biosynthesis	A, B, D, E, F
Glycosphingolipid biosynthesis-ganglio series	A, B, D
Arachidonic acid metabolism	A, B
Terpenoid backbone biosynthesis	A, B
Zeatin biosynthesis	A, B
Cutin, suberine and wax biosynthesis	A, D, E, F
Photosynthesis-antenna proteins	A, E, F, G
Phenylalanine metabolism	A, G
Prodigiosin biosynthesis	B, F, G
Anthocyanin biosynthesis	B
Fructose and mannose metabolism	C, D, E, F, G
Sphingolipid metabolism	C, D, F
Fatty acid biosynthesis	C, F, G
Inositol phosphate metabolism	C, D
Pyruvate metabolism	C, G
Phosphatidylinositol signaling system	C
Glycosaminoglycan degradation	A, C, D
Plant-pathogen interaction	B, C, E, F, G
Circadian rhythm-plant	B, C, D
Thiamine metabolism	D, E, F.
Glycerophospholipid metabolism	D, E
Ubiquinone and other terpenoid−quinone biosynthesis	D, F
Flavonoid biosynthesis	E
Linoleic acid metabolism	E
Indole alkaloid biosynthesis	E, G
Other types of O−glycan biosynthesis	F
Ascorbate and aldarate metabolism	G
Protein export	G
Riboflavin metabolism	G
Lysine biosynthesis	G
Valine, leucine and isoleucine biosynthesis	G

A: Samples collected from FD across four developmental stages. B: Samples collected from MU across four developmental stages. C: Samples collected from LH across four developmental stages. D: Samples collected from BS stage across three genotypes. E: Samples collected from IF stage across three genotypes. F: Samples collected from FB stage across three genotypes. G: Samples collected from DE stage across three genotypes.

#### 3.3.4 Identification of DEGs encoding transcription factors

Transcription factors (TFs), a kind of DNA-binding proteins which play important roles in transcription, perform a number of function in flowering (Kumar et al., 2012; Shibuya et al., 2015). In total, 116 differentially expressed TFs, belonging to 26 TF families including *bHLH*, *C2H2*, *ERF*, *B3*, *MYB-related*, *NAC*, *HD-ZIP*, *bZIP*, *GRAS*, *HSF*, *MYB*, *Trihelix*, etc., were identified across the four flower developmental stages in the tree peony varieties. Among the above identified TFs, the *bHLH* family, *C2H2* family and *ERF* family accounted for the largest proportion, the following are *B3* family, *MYB*-related family protein and *NAC* family (**Table S7**).

In addition, 1,117 differentially expressed TFs belonging to 53 TF families, covering *bHLH*, *ERF*, *NAC*, *C2H2*, *B3*, *MYB*-related, *Trihelix*, *MYB*, *FAR1*, *GRAS*, *C3H*, and *WRKY*, etc., were identified across the three varieties at BS, IF, FB, and DE stages. Among these TFs, the *bHLH* family accounted for the largest proportion, followed by *ERF*, *NAC*, *C2H2*, *B3*, and *MYB*-related family (**Table S8**). It was worth mention that *bHLH* family, *C2H2* family, *ERF* family, *B3* family, *MYB*-related family, and *NAC* family showed high dominance both across genotype and across developmental stages, which demonstrated that these TFs might play crucial functions in regulating floral florescence, development and senescence in tree peony.

### 3.4 Degradome sequencing revealed miRNA-regulated mRNAs

Degradome, used for miRNA and siRNA targets characterization by the 5’-ends of uncapped RNAs (German et al., 2008; Gregory et al., 2008). Petals of the four flower developmental stages (BS, IF, FB and DE) for each genotype (FD, MU and LH) were pooled prior the degradome libraries construction (FD, LH, and MU) and used for degradome analysis. Around 10.9 million raw reads were gained from the degradome libraries. Detailed information of total raw reads, unique raw reads, mappable reads, unique mappable reads, mapped reads, unique mapped reads, number of input transcripts, and number of target transcripts are shown in **Table S9**. A total of 7,571, 9,250 and 6,457 mRNAs were silenced by miRNAs in FD, MU and LH respectively. The alignment information of miRNAs-mRNA pairs can be found in **Table S10**. In total, 11,391 targets showed a differential degradation pattern between FD and LH. In addition, 11,928 targets presented a differential degradation characteristic between MU and FD. Moreover 11,605 targets demonstrated a differential degradation characteristic between MU and LH.

### 3.5 Integrated analysis of floral florescence, development, and senescence dependent miRNA-mRNA modules

DEGs identified across flower developmental stages (BS, IF, FB, DE) in FD, MU and LH were assembled into a unified set. DEGs identified across varieties (FD, MU, LH) at developmental stages BS, IF, FB and DE were then assembled into another unified set. DEGs from the union sets were then used together for further miRNA-mRNA target pairing confirmation by multi-omics analysis. Subsequently, miRNA-mRNA pairs with opposite regulatory patterns were characterized in terms of the gene-silencing function of miRNAs. With this method, miRNA-mRNA pairs associated with floral florescence, development and senescence were identified.

**Figure 6:**
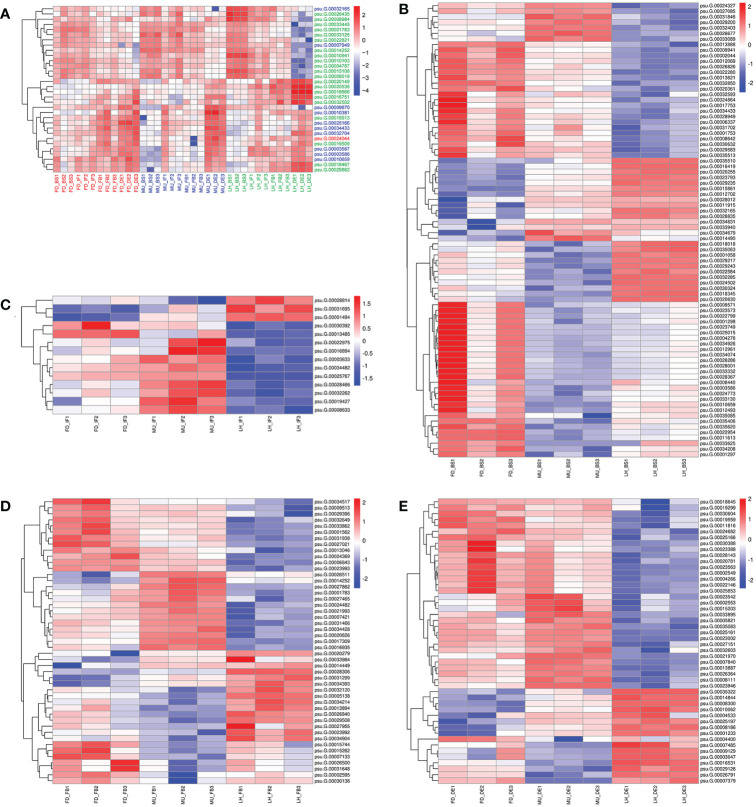
Expression pattern of miRNA targets specific to flower developmental stages and varieties by miRNAome, transcriptome and degradome integrated analysis of tree peony. **(A)** Expression pattern of miRNA targets identified across flower developmental stages in FD, MU, and LH. **(B)** Expression pattern of miRNA targets identified across tree peony varieties at flower developmental stage BS. **(C)** Expression pattern of miRNA targets identified across tree peony varieties at flower developmental stage IF. **(D)** Expression pattern of miRNA targets identified across tree peony varieties at flower developmental stage FB. **(E)** Expression pattern of miRNA targets identified across tree peony varieties at flower developmental stage DE.

**Figure 7:**
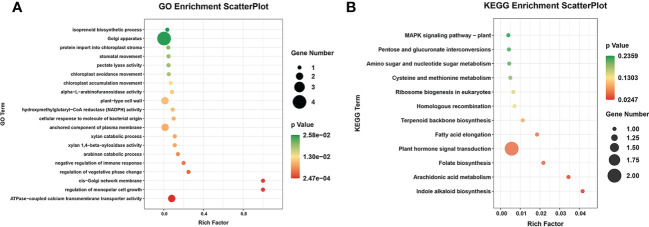
GO and KEGG analysis of miRNA targets specific to flower developmental stages and varieties by miRNAome, transcriptome and degradome integrated analysis of tree peony. **(A)** GO analysis of target genes identified by integrated analysis of miRNAome, transcriptome and degradome. **(B)** KEGG pathway analysis of target genes identified by integrated analysis of miRNAome, transcriptome and degradome.

In total, 32 miRNA targets showed antagonistic regulatory patterns during developmental stages in FD, MU and LH, which might thus be candidates for regulating the floral development and senescence of tree peony (**Table S11**). Another 191 miRNA targets were differentially expressed across tree peony varieties and developmental stages, which suggests important roles for those in floral florescence regulation (i.e., the timing of flower opening and senescence) (**Table S11**). Of these, ten miRNA targets showed significantly different expression patterns both across development and across varieties, which suggests possible dual roles in floral florescence regulation in tree peony. Expression patterns of these floral florescence, development and senescence dependent miRNA targets are presented in **Figures 6A–E**. GO (**Figure 7A**) and KEGG (**Figure 7B**) pathway analysis revealed that these floral florescence-, development- and senescence-dependent miRNA targets were enriched in pathways like plant hormone signal transduction, indole alkaloid biosynthesis, arachidonic acid metabolism, folate biosynthesis, fatty acid elongation, MAPK signaling pathway, etc.

**Figure 8:**
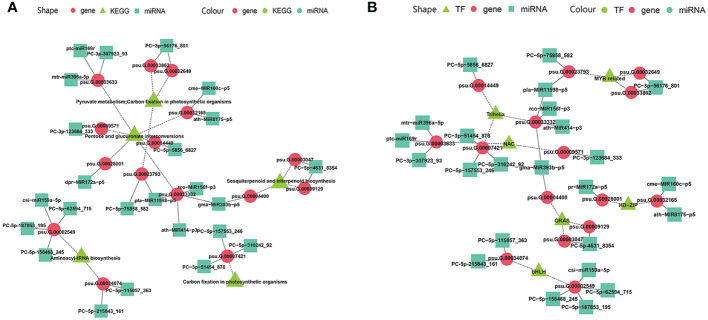
MiRNA-mRNA-TF modules regulatory network identified by miRNAome, transcriptome and degradome integrated analysis across flower developmental stages and varieties of tree peony **(A)** Regulatory network mediated by miRNA-mRNA-TF modules identified across flower developmental stages in FD, MU, and LH. **(B)** Regulating network mediated by miRNA-mRNA-TF modules identified across tree peony varieties at flower developmental stages BS, IF, FB, DE.

The miRNA-guided floral florescence, development and senescence regulatory networks were complicated, which imply that one specific miRNA might be able to adjust and control many mRNAs, and one specific mRNA also might be targeted by divers miRNAs simultaneously (Liu et al., 2020a). Multiple-to-multiple inter-associations between miRNAs and their target genes which enriched in KEGG pathways and encode TFs were constructed by Cytoscape (**Figure 8**). The multiple-to-multiple miRNA-mRNA-TF modules were enriched in pyruvate metabolism, carbon fixation in photosynthetic organisms, pentose and glucuronate interconversions, sesquiterpenoid and triterpenoid biosynthesis, aminoacyl-tRNA biosynthesis (**Figure 8A**). The result also displayed that the miRNA-mRNA-TF modules mainly consisted of *MYB*-related, *bHLH*, *Trihelix*, *NAC*, *GRAS* and *HD-ZIP* TF families, demonstrating their potential functions in tree peony floral florescence, development and senescence (**Figure 8B**).

### 3.6 Weighted gene co-expression network analysis reveals candidate hub genes

**Figure 9:**
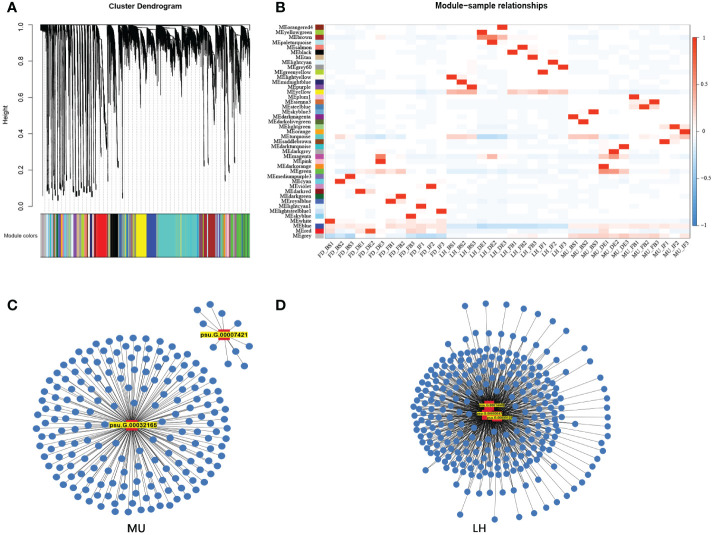
WGCNA analysis of differentially expressed genes regulating floral florescence, development and senescence. **(A)** Hierarchical cluster tree showing co-expression modules. **(B)** Module-samples relationships. **(C)** Hub gene regulating floral florescence, development and senescence in LH. **(D)** Hub gene regulating floral florescence, development and senescence in MU.

In order to uncover the regulation mechanism of flowering time based on transcriptome data across varieties and flower development stages, weighted gene co-expression network analysis (WGCNA) was performed to detect co-expressed genes to disclose the hub gene which might regulate floral florescence, development and senescence. WGCNA analysis resulted in 43 distinct co-expressed gene modules were exhibited by distinctive colors and shown by a heatmap (**Figure 9A**). Each heatmap represented an expression cluster, which straightly elucidated the relationship between the clusters of three tree peony varieties and four development stages (**Figure 9B**). Then, correlation analysis was performed between modules and samples to find modules with the highest correlation. The candidate hub genes were confirmed by taking the intersection of gene in modules with the highest correlation and the genes used for integrated analysis of miRNA-mRNA-TF. Furthermore, the intersected genes were selected for the hub genes network construction (**Figures 9C, D**). Finally, hub genes *psu.G.00014449*, *psu.G.00003047* and *psu.G.00009129* in LH, *psu.G.00032165* and *psu.G.00007421* in MU may play crucial roles in the floral florescence, development and senescence in tree peony. Regrettably, none intersected hub genes were detected in FD since the lower gene connectivity.

### 3.7 qRT-PCR analysis for the DEMs and DEGs verification

**Figure 10:**
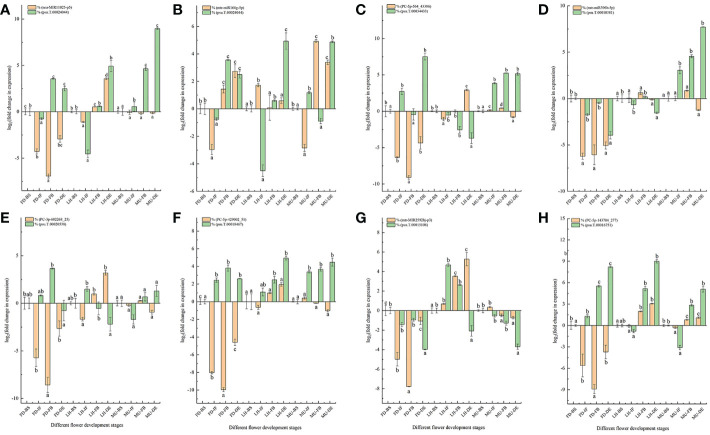
qRT-PCR analysis of miRNA-mRNA pairs. **(A)** qRT-PCR analysis of *MIR11025-p5* and *psu.T.00024044*. **(B)** qRT-PCR analysis of *miR166-5p* and *psu.T.00024044*. **(C)** qRT-PCR analysis of *PC-5p-564_43386* and *psu.T.00034433*. **(D)** qRT-PCR analysis of *mtr-miR396b-5p* and *psu.T.00010381*. **(E)** qRT-PCR analysis of *PC-3p-602268_25* and *psu.T.00020538*. **(F)** qRT-PCR analysis of *PC-5p-429002_51* and *psu.T.00018467*. **(G)** qRT-PCR analysis of *mtr-MIR2592bj-p3* and *psu.T.00015108*. **(H)** qRT-PCR analysis of *PC-5p-143784_277* and *psu.T.00016751*. MU, Mutant of Paeonia ostii ‘Fengdan’; FD, Paeonia ostii ‘Fengdan’; LH, Paeonia suffruticosa ‘Lianhe’; BS, Blooming Stage; IF, Initial Flowering Stage; FB, Full Blooming Stage; DE, Decay Stage.

A total of eight DEM and DEG pairs were selected for the qRT-PCR analysis to verify the expression pattern of miRNA and mRNA data obtained from miRNAome, transcriptome, and degradome (**Figure 10**). The selected miRNA-target pairs were presented as follows: *seu-MIR11025-p5* and *psu.T.00024044*, *miR166-5p* and *psu.T.00024044*, *PC-5p-564_43386* and *psu.T.00034433*, mtr-*miR396b-5p* and *psu.T.00010381*, *PC-3p-602268_25_S* and *psu.T.00020538*, *PC-5p-429002_51_S* and *psu.T.00018467*, *mtr-MIR2592bj-p3* and *psu.T.00015108*, *PC-5p-143784_277_S* and *psu.T.00016751*. Target t-plots (**Figure S6**) show the cleavage sites of target genes silenced by miRNAs during developmental stages. In general, the expression levels of DEM-target pairs were consistent with miRNA-guided mRNA cleavage signatures validated by degradome.

As expected, majority of the expression patterns of the examined miRNA-target pairs were similar to the RNA-seq data, with the exception of transcripts *psu.T.00010381*, *psu.T.00016751* and *mtr-miR396b-5p*, confirming the accuracy and reliability of the sequencing data in general (**Figure 10**). Our study confirmed an exactly negative correlation for miRNAs and target genes *PC-3p-602268_25* and *psu.T.00020538* expressed at the four developmental stages in FD and LH, target genes *PC-5p-429002_51* and *psu.T.00018467* at all four developmental stages in FD, and target genes *mtr-miR166g-5p* and *psu.T.00024044* at the four developmental stages in MU. While the remaining tested miRNA-target pairs did not always show a negative relationship across the four developmental stages, this is consistent with previous research (Liu et al., 2017; Zuluaga et al., 2017).

Previous research revealed that miRNA and their target genes were not always presented a specific one-to-one regulatory relationship, which is due to that a specific miRNA can regulate several target mRNAs simultaneously and a specific mRNA also could be targeted by multiple miRNAs (Liu et al., 2020a; Liu et al., 2020b). Thus, the expression pattern of miRNA and their target genes does not emerge a negative correlation all the time (Liu et al., 2017; Zuluaga et al., 2017; Zuluaga et al., 2018). Our study showed that *psu.T.00024044* could be targeted by *mtr-miR166g-5p* and *seu-MIR11025-p5_2ss4CA17CA* simultaneously which was consistent with data elucidated previously. Furthermore, changes in expression levels of miRNA and mRNA could have derived from the differences arising from the biological replicates (Pradervand et al., 2009; Xu et al., 2018; Zuluaga et al., 2018; Liu et al., 2020b). Correlation analysis of miRNA-target pairs expression profiles between sequencing and qRT-PCR are shown in **Figure S7**.

## 4 Discussion

Florescence plays a crucial role in ornamental value of tree peony. However, current understanding of the regulatory mechanism underlying florescence in tree peony is still far beyond understanding. Here, we reported the combined analysis of the miRNAome, transcriptome and degradome to reveal the potential regulatory mechanism of florescence, using petals from four developmental stages in three tree peony varieties-FD (an early-flowering variety), LH (a late-flowering variety), and MU (a natural mutant line of FD) that flowers 2-3 days earlier than FD. Previous research on tree peony miRNA discovery sampled bud (Zhang et al., 2018; Han et al., 2020) or root, stem and leaf (Jin et al., 2015) tissues of FD, as well as seeds of high and low-ALA-content varieties *Paeonia rokii* ‘Sai gui fei’ and ‘Jing shen huan fa’ (Hao et al., 2017). This is also the first report of miRNA-target identification in varieties with contrasting flowering time phenotypic trait. Knowledge obtained on the miRNA-mRNA modules in tree peony varieties will provide crucial preliminary data for the further researches on floral florescence, development and abscission in tree peony.

### 4.1 Pathways associated with floral florescence, development, and senescence

Floral-associated pathways usually involved the vernalization, autonomous, ambient temperature, photoperiod, gibberellic acid, aging and sugar pathways (Han et al., 2021). Genes involved flowering-time regulation in tree peony (Wang et al., 2019) that were identified including genes involved in floral organ and meristem, vernalization pathway, age pathway, GA pathway, autonomous pathway, photoperiod pathway. High intensity light promotes flowering through the photoperiod pathway which requires the cooperation of chloroplast retrograde signals and silencing transcription of *Flowering Locus C* (*FLC*). To response high light induction, transcription factor *PTM* localized at chloroplast envelope suppresses *FLC* transcription. It is also known that an intracellular signaling pathway originated from chloroplasts regulates the flowering transition (Feng et al., 2016; Susila et al., 2016). Furthermore, it was discovered that the expression of chloroplast protein *CEBP* changed during flower development and senescence (Iordachescu et al., 2009). Our results showing that chloroplast and chloroplast envelope genes were expressed in common among both varieties and developmental stages assayed, suggests important roles in floral florescence, development and senescence in tree peony. We also observed a general role for the interaction of turgor pressure and plasmodesmata affecting floral development, perhaps through regulation of plasmodesmata aperture, for which an association with transition to flowering was previously shown (Hernández-Hernández et al., 2019).

### 4.2 Plant hormone signal transduction contributing to floral florescence, development, and senescence

Cell division, expansion, differentiation and stress response in organisms were proved to be regulated by hormones in the earlier report (Artur, 2022). ABA signaling presents multiple connections with the photoperiodic pathway (Martignago et al., 2020). Exogenous spraying of ABA result in changes of flowering time, indicating that ABA possibly is an internal factor regulating the floral transition (Conti et al., 2014). *GIGANTEA* (*GI*) is a key flowering gene required for photoperiod perception (Mishra and Panigrahi, 2015). ABA signaling integration through *GI* operates *via* up-regulation of *FT* (Riboni et al., 2016). Overexpression of the chrysanthemum *R2R3-MYB* delays flowering in Arabidopsis (Shan et al., 2012). ABA hypersensitive 1 suppresses frigida-mediated delayed flowering in Arabidopsis (Bezerra et al., 2004). *FD* and *FD-like bZIPs* protein complexes play a significant role in modulating ABA signaling (Martignago et al., 2020). ABA activates an intricate regulatory network of signals including TFs that have contrary effects on florescence (Conti et al., 2014). While the role of ABA in flowering in model plants is emerging, the ABA molecular control of flowering still poorly revealed in tree peony. This research found that many of the most highly differentially expressed genes were relevant to plant hormone signal transduction especially ABA. These results might provide a potential basis for further research on mechanisms parsing of ABA regulating floral florescence, development and senescence in tree peony.

### 4.3 miRNA-mRNA-TF regulate floral florescence, development, and senescence

MiRNAs have been proved to be of great importance in regulation of gene expression, defense responses, and cell function in plants (Achkar et al., 2016; Choudhary et al., 2021). Recent research have shown that miRNAs play crucial roles in regulating gene expression associated with flowering (Spanudakis and Jackson, 2014). According to the latest report, *miR156* and *miR172* possibly participate in flowering *via* an aging pathway (Waheed and Zeng, 2020). *MiR167* was reported to be involved in governing floral/fiber-associated agronomic traits in cotton (Arora and Chaudhary, 2021). Inhibition of *miR168* in rice could improve yield, prolong flowering and enhance immunity (Wang et al., 2021). While overexpression of *miR159* resulted in late flowering, whereas suppression of *miR159* leaded to the acceleration of flowering in the ornamental flowering plant gloxinia (Li et al., 2013; Millar et al., 2019).

Researches have demonstrated that TFs play vital roles in floral transition as miRNA targets. MiRNAs and their TF targets regulate gene expression at post-transcriptional level and transcriptional level respectively (Waheed and Zeng, 2020). Previously, the miRNA-mRNA-TF modules like *pos-miR319a-3p.2–3p/TCP2*, *pos-miR159/GAMYB*, *pos-miR169/nuclear transcription factor Y subunit A*, and *pos-miR828/WER* were identified in variety FD (Han et al., 2020). TF target genes (*AP2* and *SPL*) might have splice sites for *PsmiR172a* and *PsmiR156a*, suggesting that *miR156* and *miR172* probably play important roles during dormancy transition in FD (Zhang et al., 2018). Additionally, studies have revealed that *miR156-SPL* (Xie et al., 2020; Rao et al., 2021), *miR172-AP2* (Ó’Maoiléidigh et al., 2021), *miR319-TCP* (Zhu et al., 2021), *miR159-MYB* (Spanudakis and Jackson, 2014) and *miR399-PHO2* (Kim et al., 2011) play important roles in floral transition.

Transcription factors *MYC2*, *MYC3*, and *MYC4* in *bHLH* family were involved in jasmonate-mediated flowering inhibition in Arabidopsis (Wang et al., 2017). Previous studies showed that the *bZIP* transcription factors were functionally required for flower development (Strathmann et al., 2001). The *TBZF* gene encoding *bZIP* were reported to be abundant in senescing flower buds (Yang et al., 2002). The *C2H2* zinc finger family perform functions in pollen development regulation in grapevine (Arrey-Salas et al., 2021). Overexpression of the *CcNAC1* gene promotes early flowering in jute (Zhang et al., 2021). Acting as a *B3* domain transcription factor, *AtREM16* prolongs flowering by coupling on the promoters of *SOC1* and *FT* (Yu et al., 2020). Expression of chrysanthemum *Trihelix* transcription factors showed that they played important roles in chrysanthemum inflorescences (Song et al., 2016). It has also been shown that expression of chrysanthemum transcription factor *ERF* can influence flowering time in Arabidopsis (Xing et al., 2019). Additionally, it was shown that *CmERF110* interacts with *CmFLK* to promote flowering by regulating the circadian clock (Huang et al., 2022).

In this study, we identified 16 miRNA-mRNA-TF modules across flower developmental stages (**Table S12**). The TFs belongs to 16 families. Among these TF families, the *bHLH*, *bZIP*, and *C2H2* accounted for the largest proportion, followed by *B3*, *Trihelix*, *ERF*, etc. In addition, 71 miRNA-mRNA-TF modules were identified across the tree peony varieties at the flower developmental stages (**Table S12**). The TFs consisted of 37 TF families, of which the *NAC*, *Trihelix* and *bHLH* families accounted for the largest proportion, followed by *ERF*, *B3*, *MYB* family etc. In this study, the *bHLH*, *NAC*, *C2H2*, *bZIP*, and *Trihelix* displayed the most highly differential expression, suggesting that these miRNA-mRNA-TF modules may be crucial factors in floral florescence, development and senescence in tree peony. Their specific functional contributions in tree peony remains to be further explored.

### 4.4 Limiting factors in the study and future research on tree peony

The first draft genome assembly (~13.79 GB) of tree peony variety ‘Luo shen xiao chun’ reported recently represents the largest sequenced genome in dicotyledon to date (Lv et al., 2020). However, due to the unusually large and complex genome, the draft genome assembly of tree peony is still on scaffold level, which hinders miRNA-mRNA pairs identification by transcriptome and sRNAome analysis using the reference genome. A high-quality reference genome is expected to provide substantial fundamental resources for further research in tree peony in the future. Furthermore, lack of a homologous genetic transformation system has hindered functional genomics research in tree peony (Wen et al., 2020). Breakthroughs in virus-induced gene silencing (VIGS) in rose (Cheng et al., 2018; Liang et al., 2020; Cheng et al., 2021; Zhang et al., 2021) and tree peony (Zhao et al., 2017; Xie et al., 2019; Wang et al., 2020; Han et al., 2022), provide hope for the functional characterization and identification of miRNA-mRNA modules for floral florescence, development, and senescence identified in this study in tree peony.

## 5 Conclusion

Prolonging blooming period has been an important target for tree peony breeding. The present study provides the integrated analysis of tree peony miRNA-mRNA modules regulated at the transcriptional level, with the purpose of illustrating the regulatory network of floral florescence, development, and senescence. The expression profiles described include developing flowers at multiple developmental time-points, in three varieties with contrasting flowering time (early- and late-flowering), including an early-flowering mutant line found previously. A total of 2,444 tree peony miRNAs were identified, with 1,709 of them being novel. A transcriptome analysis resulted in discovery of 35,687 genes, with a significant number of floral florescence, development, and senescence DEGs associated with chloroplast, chloroplast envelope, plasmodesma, and molecular function process, and involved in plant hormone signal transduction, starch and sucrose metabolism, MAPK signaling, phenylpropanoid and carotenoid biosynthesis pathway. Multi-omics analysis of flowering time regulation networks identified key miRNA-target pairs including transcription factors, protein kinases, and hormone regulators that were antagonistically regulated. Newly discovered functional miRNA-mRNA-TF modules provide molecular resources for further interpretation of the florescence mechanism, and for germplasm resource innovation aimed at prolonging flowering time of tree peony.

## Data availability statement

The data presented in the study are deposited in the China National GenBank repository, accession number CNP0002984, https://db.cngb.org/search/?q=CNP0002984.

## Author contributions

LG performed the experiments, analyzed the data and wrote the manuscript. YL, CZ, and ZW participated in the sample collection and experimental assay. JC and WY contributed to the manuscript review and editing. XZ contributed to the experimental design, review and editing. XH contributed to the conceptualization, review, editing, and supervision. All authors contributed to the article and approved the submitted version.

## Supplementary material

Supplementary Figure 1 Expressed miRNA detected across flower developmental stages and varieties in tree peony. **(A)** The distribution of expressed miRNAs across the four flower developmental stages (BS, IF, FB, DE) in FD. **(B)** The distribution of expressed miRNAs across the four flower developmental stages (BS, IF, FB, DE) in MU. **(C)** The distribution of expressed miRNAs across the four flower developmental stages (BS, IF, FB, DE) in LH. **(D)** Intersection of expressed miRNAs across flower developmental stages (BS, IF, FB, DE) and tree peony varieties (LH, MU and LH). **(E)** The distribution of expressed miRNAs across varieties (FD, MU and LH) at flower developmental stage BS. **(F)** The distribution of expressed miRNAs across varieties (FD, MU and LH) at flower developmental stage IF. **(G)** The distribution of expressed miRNAs across varieties (FD, MU and LH) at flower developmental stage FB. **(H)** The distribution of expressed miRNAs across varieties (FD, MU and LH) at flower developmental stage DE. **(I)** Intersection of expressed miRNAs across tree peony varieties (FD, MU and LH) and flower developmental stages (BS, IF, FB, DE).

Supplementary Figure 2 Summary of the number of miRNAs showing significant differential expression at different flower developmental stages in FD, MU, and LH. **(A)** Number of miRNAs showing significant differential expression (*P*< 0.01, *P*<0.05, and *P*<0.1) at different flower developmental stages in FD. **(B)** Number of miRNAs showing significant differential expression (*P*< 0.01, *P*<0.05, and *P*<0.1) at different flower developmental stages in MU. **(C)** Number of miRNAs showing significant differential expression (*P*< 0.01, *P*<0.05, and *P*<0.1) at different flower developmental stages in LH. The blue colored bars represent the number of upregulated miRNAs. The red colored bars represent the number of downregulated miRNAs.

Supplementary Figure 3 Annotated genes detected across flower developmental stages in FD, MU, and LH. **(A)** The distribution of annotated genes across developmental stages (BS, IF, FB, DE) in tree peony genotype FD. **(B)** The distribution of annotated genes across developmental stages (BS, IF, FB, DE) in tree peony genotype MU. **(C)** The distribution of annotated genes across developmental stages (BS, IF, FB, DE) in tree peony genotype LH. **(D)** Intersection of annotated genes across developmental stages (BS, IF, FB, DE) and tree peony varieties (LH, MU and LH).

Supplementary Figure 4 Annotated genes detected across tree peony varieties at different flower developmental stages. **(A)** The distribution of annotated genes across varieties (FD, MU and LH) at developmental stage BS. **(B)** The distribution of annotated genes across varieties (FD, MU and LH) at developmental stage IF. **(C)** The distribution of annotated genes across varieties (FD, MU and LH) at developmental stage FB. **(D)** The distribution of annotated genes across varieties (FD, MU and LH) at developmental stage DE. **(E)** Intersection of annotated genes across varieties (FD, MU and LH) and flower developmental stages (BS, IF, FB, DE).

Supplementary Figure 5 Summary of the number of genes showing significant differential expression at different flower developmental stages in FD, MU, and LH. **(A)** Number of genes showing significant differential expression (*P*<0.05) across different flower developmental stages (BS, IF, FB, DE) in tree peony variety FD, MU, and LH, respectively. **(B)** Number of genes showing significant differential expression (*P*<0.05) across varieties (FD, MU, LH) at different flower developmental stages (BS, IF, FB, DE), respectively. The blue colored bars represent the number of upregulated genes. The red colored bars represent the number of downregulated genes.

Supplementary Figure 6 T plot of mRNA-miRNA pairs selected and verified by qRT-PCR. **(A)** T plot of *MIR11025-p5* and *psu.T.00024044*. **(B)** T plot of *miR166-5p* and *psu.T.00024044*. **(C)** T plot of *PC-5p-564_43386* and *psu.T.00034433*. **(D)** T plot of *mtr-miR396b-5p* and *psu.T.00010381*. **(E)** T plot of *PC-3p-602268_25_S* and *psu.T.00020538*. **(F)** T plot of *PC-5p-429002_51_S* and *psu.T.00018467*. **(G)** T plot of *mtr-MIR2592bj-p3* and *psu.T.00015108*. **(H)** T plot of *PC-5p-143784_277_S* and *psu.T.00016751*.

Supplementary Figure 7 Correlation analysis of miRNA and target expression profiles between high-throughput sequencing and qRT-PCR. **(A)** Correlation analysis of *psu.T.00024044* with *MIR11025-p5* and *miR166-5p*. **(B)** Correlation analysis of *psu.T.00034433* with *PC-5p-564_43386.* **(C)** Correlation analysis of *psu.T.00010381* with *mtr-miR396b*-5p. **(D)** Correlation analysis of *psu.T.00020538* with *PC-3p-602268_25*. **(E)** Correlation analysis of *psu.T.00018467* with *PC-5p-429002_51*. **(F)** Correlation analysis of *psu.T.00015108* with *mtr-MIR2592bj-p3*. **(G)** Correlation analysis of *psu.T.00016751* with *PC-5p-143784_277*.
