# Supplementary material for: Integrated analysis of miRNAome transcriptome and degradome reveals miRNA-target modules governing floral florescence development and senescence across early- and late-flowering genotypes in tree peony
Source: Front Plant Sci. 2022 Dec 14;13:1082415. doi: 10.3389/fpls.2022.1082415 (PMC9795019; doi:10.3389/fpls.2022.1082415)
Supplement: Supplementary Figure 1 — Expressed miRNA detected across flower developmental stages and varieties in tree peony. (A) The distribution of expressed miRNAs across the four flower developmental stages (BS, IF, FB, DE) in FD. (B) The distribution of expressed miRNAs across the four flower developmental stages (BS, IF, FB, DE) in MU. (C) The distribution of expressed miRNAs across the four flower developmental stages (BS, IF, FB, DE) in LH. (D) Intersection of expressed miRNAs across flower developmental stages (BS, IF, FB, DE) and tree peony varieties (LH, MU and LH). (E) The distribution of expressed miRNAs across varieties (FD, MU and LH) at flower developmental stage BS. (F) The distribution of expressed miRNAs across varieties (FD, MU and LH) at flower developmental stage IF. (G) The distribution of expressed miRNAs across varieties (FD, MU and LH) at flower developmental stage FB. (H) The distribution of expressed miRNAs across varieties (FD, MU and LH) at flower developmental stage DE. (I) Intersection of expressed miRNAs across tree peony varieties (FD, MU and LH) and flower developmental stages (BS, IF, FB, DE). [file DataSheet_1.zip › Supplymentary files/Supplementary tables/Table S2 Kits and reagents used for libraries construction of miRNAome.docx]

Table S2 Kits and reagents used for libraries construction of miRNAome, transcriptome and degradome

| Reaction | Kit nd reagent used for reaction |
| --- | --- |
| Total RNA extraction for transcriptome | Total RNA Purification Kit (LC Sciences, Houston, USA) |
| Small RNA library construction | TruSeq Small RNA Sample Prep Kits (Illumina, San Diego, USA) |
| Small RNA library quality evaluation | High Sensitivity DNA Chip Kit (Agilent, CA, USA) |
| Poly (A) RNA purification | Dynabeads Oligo (dT)25-61005 (Thermo Fisher, CA, USA) |
| Poly(A) RNA fragmention | Magnesium RNA Fragmentation Module (NEB, cat.e6150, USA) |
| Cleaved RNA fragments reverse transcription | SuperScript™ II Reverse Transcriptase (Invitrogen, cat. 1896649, USA) |
| U-labeled second-stranded DNA synthesis | E. coli DNA polymerase I (NEB, cat.m0209, USA), RNase H (NEB, cat.m0297, USA) and dUTP Solution (Thermo Fisher, cat. R0133, USA) |
| Single/dual-index adapters ligation and fragment size selection | AMPureXP beads |
| heat-labile UDG enzyme | NEB, cat.m0280, USA |
| Total RNA extraction for RT-qPCR | RNAprep Pure Plant Kit (TIANGEN, Beijing, China) |
| miRNA extraction for RT-qPCR | miRcute Plant miRNA Isolation Kit (TIANGEN, Beijing, China) |
| mRNA cDNA synthesis for RT-qPCR | PrimeScript cDNA Synthesis Kit (Takara, Dalian, China) |
| miRNA cDNA synthesis | miRcute Plus miRNA First-Strand cDNA Kit (TIANGEN, Beijing, China) |
| RT-qPCR for target genes | TB Green Premix Ex Taq II kit (Takara, Dalian, China) |
| RT-qPCR of miRNAs | miRcute Plus miRNA qPCR Detection Kit (TIANGEN, Beijing, China) |
