# Supplementary material for: Integrated analysis of miRNAome transcriptome and degradome reveals miRNA-target modules governing floral florescence development and senescence across early- and late-flowering genotypes in tree peony
Source: Front Plant Sci. 2022 Dec 14;13:1082415. doi: 10.3389/fpls.2022.1082415 (PMC9795019; doi:10.3389/fpls.2022.1082415)
Supplement: Supplementary Figure 1 — Expressed miRNA detected across flower developmental stages and varieties in tree peony. (A) The distribution of expressed miRNAs across the four flower developmental stages (BS, IF, FB, DE) in FD. (B) The distribution of expressed miRNAs across the four flower developmental stages (BS, IF, FB, DE) in MU. (C) The distribution of expressed miRNAs across the four flower developmental stages (BS, IF, FB, DE) in LH. (D) Intersection of expressed miRNAs across flower developmental stages (BS, IF, FB, DE) and tree peony varieties (LH, MU and LH). (E) The distribution of expressed miRNAs across varieties (FD, MU and LH) at flower developmental stage BS. (F) The distribution of expressed miRNAs across varieties (FD, MU and LH) at flower developmental stage IF. (G) The distribution of expressed miRNAs across varieties (FD, MU and LH) at flower developmental stage FB. (H) The distribution of expressed miRNAs across varieties (FD, MU and LH) at flower developmental stage DE. (I) Intersection of expressed miRNAs across tree peony varieties (FD, MU and LH) and flower developmental stages (BS, IF, FB, DE). [file DataSheet_1.zip › Supplymentary files/Supplementary tables/Table S3 RT-qPCR primers used in this study.docx]

Table S3 RT-qPCR primers used in this study

| miRNA ID/Gene ID | Annotation | Forward primer (5' to 3') | Reverse primer (5' to 3') |
| --- | --- | --- | --- |
| U6 |  | ACAGAGAAGATTAGCATGGCC | Universal primer provided in the miRcute Plus miRNA qPCR Detection Kit (SYBR Green) |
| seu-MIR11025-p5_2ss4CA17CA |  | TGCGGCTAATGAAGAGGAGGC | Universal primer provided in the miRcute Plus miRNA qPCR Detection Kit (SYBR Green) |
| mtr-miR166g-5p |  | CGGTGGGAATGTTGTCTGGCT | Universal primer provided in the miRcute Plus miRNA qPCR Detection Kit (SYBR Green) |
| PC-5p-564_43386 |  | GTTTAGCCCATTCGTGTCGCC | Universal primer provided in the miRcute Plus miRNA qPCR Detection Kit (SYBR Green) |
| mtr-miR396b-5p_R-3 |  | GCATTCCTACGGTTCCACAGCA | Universal primer provided in the miRcute Plus miRNA qPCR Detection Kit (SYBR Green) |
| PC-3p-602268_25 |  | CCTTCCGAGTCAGTGGCATA | Universal primer provided in the miRcute Plus miRNA qPCR Detection Kit (SYBR Green) |
| PC-5p-429002_51 |  | GAGCCGCCATCTTTAGTTCCTG | Universal primer provided in the miRcute Plus miRNA qPCR Detection Kit (SYBR Green) |
| mtr-MIR2592bj-p3_2ss12TC19AT |  | GGCATTCCCACTGTCGCTGTCTA | Universal primer provided in the miRcute Plus miRNA qPCR Detection Kit (SYBR Green) |
| PC-5p-143784_277 |  | GCCTGCCCTGGACGAGATTTA | Universal primer provided in the miRcute Plus miRNA qPCR Detection Kit (SYBR Green) |
| EF1-α |  | CCGCCAGAGAGGCTGCTAAT | GCAATGTGGGAAGTGTGGCA |
| psu.T.00024044 (target of seu-MIR11025-p5_2ss4CA17CA and mtr-miR166g-5p) | thaumatin-like protein 1 | CGTGAAAGGGTCAAATGGG | TCAGTTGGCGGACAAGTCG |
| psu.T.00034433 (target of PC-5p-564_43386) | 3-hydroxy-3-methylglutaryl-coenzyme A reductase | TTACATACTCCTCAACCACCGA | GAAAGAAGCGACAAAGCCAACT |
| psu.T.00010381 (target of mtr-miR396b-5p_R-3) | epidermis-specific secreted glycoprotein EP1-like | TAGTGGGTCAGTCTCTTCGGG | GATAAGGTTTCGGGGTGTTGT |
| psu.T.00020538 (target of PC-3p-602268_25) | GDSL esterase/lipase 1 | GGGAGGATATAAGAACGGTGG | TTTGATTGGAGATGGGTGTGG |
| psu.T.00018467 (target of PC-5p-429002_51) | uncharacterized protein LOC18054318 | CACCAAGAAAATCCGACCACA | CCTCCCCAGCCTCGAAATAAG |
| psu.T.00015108 (target of mtr-MIR2592bj-p3_2ss12TC19AT) | (+)-neomenthol dehydrogenase isoform X1 | AAGCAAGGATGGCCAGAGAT | TGAGTGAAGCCGGGACAGAA |
| psu.T.00016751 (target of PC-5p-143784_277) | aldo-keto reductase 1 | GTGGGTGTTGATGAGGTAGCG | TGGTTTTGTCCTTTGGAGGAG |
