# Supplementary material for: Integrated analysis of miRNAome transcriptome and degradome reveals miRNA-target modules governing floral florescence development and senescence across early- and late-flowering genotypes in tree peony
Source: Front Plant Sci. 2022 Dec 14;13:1082415. doi: 10.3389/fpls.2022.1082415 (PMC9795019; doi:10.3389/fpls.2022.1082415)
Supplement: Supplementary Figure 1 — Expressed miRNA detected across flower developmental stages and varieties in tree peony. (A) The distribution of expressed miRNAs across the four flower developmental stages (BS, IF, FB, DE) in FD. (B) The distribution of expressed miRNAs across the four flower developmental stages (BS, IF, FB, DE) in MU. (C) The distribution of expressed miRNAs across the four flower developmental stages (BS, IF, FB, DE) in LH. (D) Intersection of expressed miRNAs across flower developmental stages (BS, IF, FB, DE) and tree peony varieties (LH, MU and LH). (E) The distribution of expressed miRNAs across varieties (FD, MU and LH) at flower developmental stage BS. (F) The distribution of expressed miRNAs across varieties (FD, MU and LH) at flower developmental stage IF. (G) The distribution of expressed miRNAs across varieties (FD, MU and LH) at flower developmental stage FB. (H) The distribution of expressed miRNAs across varieties (FD, MU and LH) at flower developmental stage DE. (I) Intersection of expressed miRNAs across tree peony varieties (FD, MU and LH) and flower developmental stages (BS, IF, FB, DE). [file DataSheet_1.zip › Supplymentary files/Supplementary tables/Table S4 Overview of reads from raw data to cleaned sequences for miRNA sequencing in this study.docx]

Table S4 Overview of reads from raw data to cleaned sequences for miRNA sequencing in this study.

| Library | type | Raw reads | 3ADT&length filter | Junk reads | Rfam | mRNA | Repeats | valid reads | rRNA | tRNA | snoRNA | snRNA | other Rfam RNA | |
| --- | --- | --- | --- | --- | --- | --- | --- | --- | --- | --- | --- | --- | --- | --- |
|  |  | NA | Sequence type | Sequence type | RNA class | RNA class | RNA class | Sequence type | RNA class | RNA class | RNA class | RNA class | RNA class | |
| MU_BS1 | Total | 20615769 | 1668933 | 68356 | 477524 | 3511072 | 3812 | 15280357 | 403468 | 36233 | 8130 | 4890 | 24803 |  |
|  | % of Total | 100.00 | 8.10 | 0.33 | 2.32 | 17.03 | 0.02 | 74.12 | 1.96 | 0.18 | 0.04 | 0.02 | 0.12 |  |
|  | uniq | 4690993 | 512721 | 34167 | 5217 | 59253 | 164 | 4082496 | 3107 | 922 | 459 | 271 | 458 |  |
|  | % of uniq | 100.00 | 10.93 | 0.73 | 0.11 | 1.26 | 0.00 | 87.03 | 0.07 | 0.02 | 0.01 | 0.01 | 0.01 |  |
| MU_BS2 | Total | 21448399 | 1513779 | 77330 | 576221 | 3653656 | 3537 | 16113014 | 500295 | 32440 | 8305 | 5196 | 29985 |  |
|  | % of Total | 100.00 | 7.06 | 0.36 | 2.69 | 17.03 | 0.02 | 75.12 | 2.33 | 0.15 | 0.04 | 0.02 | 0.14 |  |
|  | uniq | 5243294 | 591456 | 41076 | 5454 | 63526 | 155 | 4544846 | 3359 | 919 | 426 | 277 | 473 |  |
|  | % of uniq | 100.00 | 11.28 | 0.78 | 0.10 | 1.21 | 0.00 | 86.68 | 0.06 | 0.02 | 0.01 | 0.01 | 0.01 |  |
| MU_BS3 | Total | 27090935 | 2133080 | 82236 | 789418 | 4989662 | 3975 | 19760003 | 684142 | 49654 | 14202 | 7019 | 34401 |  |
|  | % of Total | 100.00 | 7.87 | 0.30 | 2.91 | 18.42 | 0.01 | 72.94 | 2.53 | 0.18 | 0.05 | 0.03 | 0.13 |  |
|  | uniq | 5926679 | 761710 | 41240 | 6603 | 72389 | 192 | 5048326 | 3915 | 1191 | 616 | 369 | 512 |  |
|  | % of uniq | 100.00 | 12.85 | 0.70 | 0.11 | 1.22 | 0.00 | 85.18 | 0.07 | 0.02 | 0.01 | 0.01 | 0.01 |  |
| MU_IF1 | Total | 17969940 | 1761519 | 60847 | 600986 | 3370312 | 3471 | 12662679 | 504965 | 65291 | 7097 | 2793 | 20840 |  |
|  | % of Total | 100.00 | 9.80 | 0.34 | 3.34 | 18.76 | 0.02 | 70.47 | 2.81 | 0.36 | 0.04 | 0.02 | 0.12 |  |
|  | uniq | 3754692 | 529075 | 27149 | 5758 | 51104 | 155 | 3144976 | 3732 | 991 | 410 | 181 | 444 |  |
|  | % of uniq | 100.00 | 14.09 | 0.72 | 0.15 | 1.36 | 0.00 | 83.76 | 0.10 | 0.03 | 0.01 | 0.00 | 0.01 |  |
| MU_IF2 | Total | 16375928 | 1839293 | 56875 | 642070 | 2987803 | 2431 | 11403388 | 567250 | 37867 | 11399 | 5125 | 20429 |  |
|  | % of Total | 100.00 | 11.23 | 0.35 | 3.92 | 18.25 | 0.01 | 69.64 | 3.46 | 0.23 | 0.07 | 0.03 | 0.12 |  |
|  | uniq | 3784944 | 494088 | 28957 | 5743 | 48658 | 132 | 3210788 | 3601 | 931 | 516 | 286 | 409 |  |
|  | % of uniq | 100.00 | 13.05 | 0.77 | 0.15 | 1.29 | 0.00 | 84.83 | 0.10 | 0.02 | 0.01 | 0.01 | 0.01 |  |
| MU_IF3 | Total | 14218728 | 1284778 | 42468 | 509340 | 2548675 | 1714 | 10268066 | 445666 | 38537 | 6614 | 3144 | 15379 |  |
|  | % of Total | 100.00 | 9.04 | 0.30 | 3.58 | 17.92 | 0.01 | 72.22 | 3.13 | 0.27 | 0.05 | 0.02 | 0.11 |  |
|  | uniq | 3498052 | 436682 | 23246 | 4513 | 42850 | 106 | 2993404 | 2859 | 774 | 367 | 193 | 320 |  |
|  | % of uniq | 100.00 | 12.48 | 0.66 | 0.13 | 1.22 | 0.00 | 85.57 | 0.08 | 0.02 | 0.01 | 0.01 | 0.01 |  |
| MU_FB1 | Total | 19487728 | 914478 | 64553 | 450465 | 3451180 | 2928 | 14971157 | 385684 | 30460 | 6178 | 4414 | 23729 |  |
|  | % of Total | 100.00 | 4.69 | 0.33 | 2.31 | 17.71 | 0.02 | 76.82 | 1.98 | 0.16 | 0.03 | 0.02 | 0.12 |  |
|  | uniq | 4625111 | 512255 | 32320 | 5581 | 60393 | 154 | 4017736 | 3531 | 936 | 384 | 274 | 456 |  |
|  | % of uniq | 100.00 | 11.08 | 0.70 | 0.12 | 1.31 | 0.00 | 86.87 | 0.08 | 0.02 | 0.01 | 0.01 | 0.01 |  |
| MU_FB2 | Total | 20057655 | 730129 | 69032 | 394545 | 3363039 | 2982 | 15828806 | 342294 | 18493 | 5611 | 4361 | 23786 |  |
|  | % of Total | 100.00 | 3.64 | 0.34 | 1.97 | 16.77 | 0.01 | 78.92 | 1.71 | 0.09 | 0.03 | 0.02 | 0.12 |  |
|  | uniq | 5016613 | 485240 | 36090 | 4848 | 61424 | 149 | 4431702 | 2985 | 851 | 336 | 258 | 418 |  |
|  | % of uniq | 100.00 | 9.67 | 0.72 | 0.10 | 1.22 | 0.00 | 88.34 | 0.06 | 0.02 | 0.01 | 0.01 | 0.01 |  |
| MU_FB3 | Total | 25781737 | 2313854 | 91740 | 720656 | 4859809 | 5152 | 18398570 | 629371 | 25595 | 7869 | 6134 | 51687 |  |
|  | % of Total | 100.00 | 8.97 | 0.36 | 2.80 | 18.85 | 0.02 | 71.36 | 2.44 | 0.10 | 0.03 | 0.02 | 0.20 |  |
|  | uniq | 5812384 | 702781 | 44313 | 10683 | 85276 | 216 | 4976683 | 7909 | 1011 | 499 | 346 | 918 |  |
|  | % of uniq | 100.00 | 12.09 | 0.76 | 0.18 | 1.47 | 0.00 | 85.62 | 0.14 | 0.02 | 0.01 | 0.01 | 0.02 |  |
| MU_DE1 | Total | 16815398 | 5339056 | 56273 | 1073710 | 3582206 | 2488 | 7538104 | 830002 | 194897 | 7811 | 5869 | 35131 |  |
|  | % of Total | 100.00 | 31.75 | 0.33 | 6.39 | 21.30 | 0.01 | 44.83 | 4.94 | 1.16 | 0.05 | 0.03 | 0.21 |  |
|  | uniq | 3017314 | 669957 | 26141 | 9736 | 47258 | 151 | 2270366 | 7028 | 1358 | 339 | 282 | 729 |  |
|  | % of uniq | 100.00 | 22.20 | 0.87 | 0.32 | 1.57 | 0.01 | 75.24 | 0.23 | 0.05 | 0.01 | 0.01 | 0.02 |  |
| MU_DE2 | Total | 18084812 | 4552179 | 56894 | 1642833 | 5537151 | 3740 | 7499129 | 1347376 | 221381 | 13226 | 15588 | 45262 |  |
|  | % of Total | 100.00 | 25.17 | 0.31 | 9.08 | 30.62 | 0.02 | 41.47 | 7.45 | 1.22 | 0.07 | 0.09 | 0.25 |  |
|  | uniq | 2724551 | 590411 | 23037 | 11870 | 51612 | 180 | 2054829 | 8378 | 1674 | 591 | 413 | 814 |  |
|  | % of uniq | 100.00 | 21.67 | 0.85 | 0.44 | 1.89 | 0.01 | 75.42 | 0.31 | 0.06 | 0.02 | 0.02 | 0.03 |  |
| MU_DE3 | Total | 16804718 | 5823655 | 43084 | 1379320 | 4758047 | 3438 | 5790347 | 1113389 | 210220 | 11576 | 8874 | 35261 |  |
|  | % of Total | 100.00 | 34.65 | 0.26 | 8.21 | 28.31 | 0.02 | 34.46 | 6.63 | 1.25 | 0.07 | 0.05 | 0.21 |  |
|  | uniq | 2394133 | 587170 | 18878 | 9417 | 41341 | 174 | 1743118 | 6663 | 1325 | 463 | 300 | 666 |  |
|  | % of uniq | 100.00 | 24.53 | 0.79 | 0.39 | 1.73 | 0.01 | 72.81 | 0.28 | 0.06 | 0.02 | 0.01 | 0.03 |  |
| FD_BS1 | Total | 17862016 | 1248488 | 82359 | 511187 | 2675784 | 1964 | 13726745 | 413777 | 74449 | 4487 | 3977 | 14497 |  |
|  | % of Total | 100.00 | 6.99 | 0.46 | 2.86 | 14.98 | 0.01 | 76.85 | 2.32 | 0.42 | 0.03 | 0.02 | 0.08 |  |
|  | uniq | 5221743 | 594452 | 40738 | 6219 | 56721 | 118 | 4527381 | 4268 | 1022 | 272 | 247 | 410 |  |
|  | % of uniq | 100.00 | 11.38 | 0.78 | 0.12 | 1.09 | 0.00 | 86.70 | 0.08 | 0.02 | 0.01 | 0.00 | 0.01 |  |
| FD_BS2 | Total | 14699163 | 1963426 | 55269 | 631177 | 2771303 | 1377 | 9835155 | 581952 | 21331 | 13003 | 4171 | 10720 |  |
|  | % of Total | 100.00 | 13.36 | 0.38 | 4.29 | 18.85 | 0.01 | 66.91 | 3.96 | 0.15 | 0.09 | 0.03 | 0.07 |  |
|  | uniq | 3662088 | 473696 | 24315 | 5921 | 43144 | 92 | 3118628 | 4049 | 782 | 501 | 260 | 329 |  |
|  | % of uniq | 100.00 | 12.94 | 0.66 | 0.16 | 1.18 | 0.00 | 85.16 | 0.11 | 0.02 | 0.01 | 0.01 | 0.01 |  |
| FD_BS3 | Total | 15815658 | 1441180 | 73646 | 537220 | 2446405 | 2042 | 11774150 | 474874 | 39333 | 6843 | 3474 | 12696 |  |
|  | % of Total | 100.00 | 9.11 | 0.47 | 3.40 | 15.47 | 0.01 | 74.45 | 3.00 | 0.25 | 0.04 | 0.02 | 0.08 |  |
|  | uniq | 4558364 | 515239 | 37743 | 5033 | 47475 | 121 | 3955786 | 3260 | 859 | 358 | 220 | 336 |  |
|  | % of uniq | 100.00 | 11.30 | 0.83 | 0.11 | 1.04 | 0.00 | 86.78 | 0.07 | 0.02 | 0.01 | 0.00 | 0.01 |  |
| FD_IF1 | Total | 22289444 | 1779819 | 99550 | 700650 | 3413238 | 3101 | 16796512 | 527600 | 143287 | 6938 | 4911 | 17914 |  |
|  | % of Total | 100.00 | 7.99 | 0.45 | 3.14 | 15.31 | 0.01 | 75.36 | 2.37 | 0.64 | 0.03 | 0.02 | 0.08 |  |
|  | uniq | 5817998 | 622734 | 46697 | 7355 | 64770 | 163 | 5080646 | 4817 | 1406 | 383 | 311 | 438 |  |
|  | % of uniq | 100.00 | 10.70 | 0.80 | 0.13 | 1.11 | 0.00 | 87.33 | 0.08 | 0.02 | 0.01 | 0.01 | 0.01 |  |
| FD_IF2 | Total | 14438810 | 2041119 | 56278 | 397639 | 2209510 | 1970 | 10054972 | 337641 | 41424 | 4964 | 3432 | 10178 |  |
|  | % of Total | 100.00 | 14.14 | 0.39 | 2.75 | 15.30 | 0.01 | 69.64 | 2.34 | 0.29 | 0.03 | 0.02 | 0.07 |  |
|  | uniq | 3681459 | 447885 | 26099 | 4817 | 41816 | 110 | 3163727 | 3180 | 823 | 289 | 217 | 308 |  |
|  | % of uniq | 100.00 | 12.17 | 0.71 | 0.13 | 1.14 | 0.00 | 85.94 | 0.09 | 0.02 | 0.01 | 0.01 | 0.01 |  |
| FD_IF3 | Total | 18092713 | 4092032 | 60975 | 839717 | 3585531 | 9125 | 10195966 | 721210 | 83052 | 9266 | 4817 | 21372 |  |
|  | % of Total | 100.00 | 22.62 | 0.34 | 4.64 | 19.82 | 0.05 | 56.35 | 3.99 | 0.46 | 0.05 | 0.03 | 0.12 |  |
|  | uniq | 3380201 | 566880 | 21285 | 8896 | 50842 | 197 | 2737744 | 6077 | 1407 | 457 | 300 | 655 |  |
|  | % of uniq | 100.00 | 16.77 | 0.63 | 0.26 | 1.50 | 0.01 | 80.99 | 0.18 | 0.04 | 0.01 | 0.01 | 0.02 |  |
| FD_FB1 | Total | 13484762 | 3379818 | 41883 | 687738 | 2413663 | 2337 | 7521412 | 588852 | 78582 | 3927 | 3545 | 12832 |  |
|  | % of Total | 100.00 | 25.06 | 0.31 | 5.10 | 17.90 | 0.02 | 55.78 | 4.37 | 0.58 | 0.03 | 0.03 | 0.10 |  |
|  | uniq | 2858999 | 501538 | 19679 | 6251 | 38202 | 128 | 2297309 | 4482 | 882 | 234 | 198 | 455 |  |
|  | % of uniq | 100.00 | 17.54 | 0.69 | 0.22 | 1.34 | 0.00 | 80.35 | 0.16 | 0.03 | 0.01 | 0.01 | 0.02 |  |
| FD_FB2 | Total | 15854349 | 2551766 | 50788 | 521661 | 2952625 | 2195 | 10200344 | 447526 | 47570 | 3001 | 4840 | 18724 |  |
|  | % of Total | 100.00 | 16.10 | 0.32 | 3.29 | 18.62 | 0.01 | 64.34 | 2.82 | 0.30 | 0.02 | 0.03 | 0.12 |  |
|  | uniq | 3674149 | 479147 | 23973 | 7193 | 48660 | 137 | 3119867 | 5143 | 982 | 203 | 272 | 593 |  |
|  | % of uniq | 100.00 | 13.04 | 0.65 | 0.20 | 1.32 | 0.00 | 84.91 | 0.14 | 0.03 | 0.01 | 0.01 | 0.02 |  |
| FD_FB3 | Total | 14816500 | 1774316 | 52697 | 514244 | 2480265 | 2355 | 10402920 | 433912 | 59692 | 4676 | 3001 | 12963 |  |
|  | % of Total | 100.00 | 11.98 | 0.36 | 3.47 | 16.74 | 0.02 | 70.21 | 2.93 | 0.40 | 0.03 | 0.02 | 0.09 |  |
|  | uniq | 3627344 | 358945 | 24181 | 5791 | 45010 | 138 | 3196887 | 3954 | 946 | 307 | 196 | 388 |  |
|  | % of uniq | 100.00 | 9.90 | 0.67 | 0.16 | 1.24 | 0.00 | 88.13 | 0.11 | 0.03 | 0.01 | 0.01 | 0.01 |  |
| FD_DE1 | Total | 19042948 | 5280339 | 45268 | 986383 | 4547473 | 4113 | 8884959 | 801134 | 130261 | 6072 | 9886 | 39030 |  |
|  | % of Total | 100.00 | 27.73 | 0.24 | 5.18 | 23.88 | 0.02 | 46.66 | 4.21 | 0.68 | 0.03 | 0.05 | 0.20 |  |
|  | uniq | 3270218 | 549422 | 19409 | 9450 | 51316 | 187 | 2646546 | 6819 | 1336 | 341 | 353 | 601 |  |
|  | % of uniq | 100.00 | 16.80 | 0.59 | 0.29 | 1.57 | 0.01 | 80.93 | 0.21 | 0.04 | 0.01 | 0.01 | 0.02 |  |
| FD_DE2 | Total | 17614705 | 4897805 | 42089 | 1147359 | 4820487 | 4280 | 7520850 | 901799 | 185549 | 5247 | 7787 | 46977 |  |
|  | % of Total | 100.00 | 27.81 | 0.24 | 6.51 | 27.37 | 0.02 | 42.70 | 5.12 | 1.05 | 0.03 | 0.04 | 0.27 |  |
|  | uniq | 2955665 | 542307 | 16763 | 10107 | 47621 | 181 | 2345203 | 7403 | 1468 | 289 | 318 | 629 |  |
|  | % of uniq | 100.00 | 18.35 | 0.57 | 0.34 | 1.61 | 0.01 | 79.35 | 0.25 | 0.05 | 0.01 | 0.01 | 0.02 |  |
| FD_DE3 | Total | 21791377 | 13108678 | 23427 | 677570 | 2801320 | 4390 | 5652277 | 515174 | 131646 | 4245 | 6343 | 20162 |  |
|  | % of Total | 100.00 | 60.16 | 0.11 | 3.11 | 12.86 | 0.02 | 25.94 | 2.36 | 0.60 | 0.02 | 0.03 | 0.09 |  |
|  | uniq | 2111479 | 619011 | 9884 | 8433 | 36051 | 158 | 1443249 | 5781 | 1603 | 274 | 268 | 507 |  |
|  | % of uniq | 100.00 | 29.32 | 0.47 | 0.40 | 1.71 | 0.01 | 68.35 | 0.27 | 0.08 | 0.01 | 0.01 | 0.02 |  |
| LH_BS1 | Total | 15459309 | 2123056 | 45372 | 442373 | 1943256 | 3381 | 11281451 | 385832 | 32881 | 6822 | 3105 | 13733 |  |
|  | % of Total | 100.00 | 13.73 | 0.29 | 2.86 | 12.57 | 0.02 | 72.98 | 2.50 | 0.21 | 0.04 | 0.02 | 0.09 |  |
|  | uniq | 3621572 | 471168 | 24335 | 4204 | 36607 | 156 | 3087624 | 2668 | 633 | 363 | 213 | 327 |  |
|  | % of uniq | 100.00 | 13.01 | 0.67 | 0.12 | 1.01 | 0.00 | 85.26 | 0.07 | 0.02 | 0.01 | 0.01 | 0.01 |  |
| LH_BS2 | Total | 17422169 | 1888743 | 75082 | 251922 | 1563506 | 2993 | 13832350 | 197206 | 36124 | 4184 | 2455 | 11953 |  |
|  | % of Total | 100.00 | 10.84 | 0.43 | 1.45 | 8.97 | 0.02 | 79.40 | 1.13 | 0.21 | 0.02 | 0.01 | 0.07 |  |
|  | uniq | 5262173 | 568402 | 45983 | 3775 | 43243 | 158 | 4602801 | 2319 | 670 | 272 | 204 | 310 |  |
|  | % of uniq | 100.00 | 10.80 | 0.87 | 0.07 | 0.82 | 0.00 | 87.47 | 0.04 | 0.01 | 0.01 | 0.00 | 0.01 |  |
| LH_BS3 | Total | 18963162 | 1612772 | 69323 | 401432 | 2069857 | 3041 | 15138213 | 340743 | 34999 | 6024 | 3612 | 16054 |  |
|  | % of Total | 100.00 | 8.50 | 0.37 | 2.12 | 10.92 | 0.02 | 79.83 | 1.80 | 0.18 | 0.03 | 0.02 | 0.08 |  |
|  | uniq | 5390612 | 588386 | 40667 | 4297 | 48395 | 151 | 4711301 | 2704 | 695 | 334 | 225 | 339 |  |
|  | % of uniq | 100.00 | 10.92 | 0.75 | 0.08 | 0.90 | 0.00 | 87.40 | 0.05 | 0.01 | 0.01 | 0.00 | 0.01 |  |
| LH_IF1 | Total | 19016257 | 2719738 | 59888 | 461473 | 2231463 | 3080 | 13915387 | 387077 | 46593 | 6027 | 4138 | 17638 |  |
|  | % of Total | 100.00 | 14.30 | 0.31 | 2.43 | 11.73 | 0.02 | 73.18 | 2.04 | 0.25 | 0.03 | 0.02 | 0.09 |  |
|  | uniq | 4464344 | 539394 | 32224 | 5328 | 46573 | 152 | 3843886 | 3446 | 871 | 359 | 258 | 394 |  |
|  | % of uniq | 100.00 | 12.08 | 0.72 | 0.12 | 1.04 | 0.00 | 86.10 | 0.08 | 0.02 | 0.01 | 0.01 | 0.01 |  |
| LH_IF2 | Total | 19608933 | 3109740 | 57166 | 458532 | 2632387 | 3485 | 13689539 | 366522 | 61803 | 5440 | 6311 | 18456 |  |
|  | % of Total | 100.00 | 15.86 | 0.29 | 2.34 | 13.42 | 0.02 | 69.81 | 1.87 | 0.32 | 0.03 | 0.03 | 0.09 |  |
|  | uniq | 4529810 | 602555 | 29952 | 6472 | 50052 | 166 | 3844501 | 4251 | 1104 | 311 | 332 | 474 |  |
|  | % of uniq | 100.00 | 13.30 | 0.66 | 0.14 | 1.10 | 0.00 | 84.87 | 0.09 | 0.02 | 0.01 | 0.01 | 0.01 |  |
| LH_IF3 | Total | 17526698 | 2326406 | 57229 | 403366 | 2138211 | 3215 | 12897332 | 310411 | 70047 | 5428 | 4025 | 13455 |  |
|  | % of Total | 100.00 | 13.27 | 0.33 | 2.30 | 12.20 | 0.02 | 73.59 | 1.77 | 0.40 | 0.03 | 0.02 | 0.08 |  |
|  | uniq | 4147527 | 445618 | 30038 | 4947 | 44290 | 151 | 3625343 | 3095 | 932 | 324 | 252 | 344 |  |
|  | % of uniq | 100.00 | 10.74 | 0.72 | 0.12 | 1.07 | 0.00 | 87.41 | 0.07 | 0.02 | 0.01 | 0.01 | 0.01 |  |
| LH_FB1 | Total | 14315830 | 3208204 | 42568 | 366463 | 1820077 | 4038 | 9155403 | 292090 | 51599 | 4798 | 3352 | 14624 |  |
|  | % of Total | 100.00 | 22.41 | 0.30 | 2.56 | 12.71 | 0.03 | 63.95 | 2.04 | 0.36 | 0.03 | 0.02 | 0.10 |  |
|  | uniq | 3140763 | 508616 | 20558 | 5478 | 35716 | 151 | 2573629 | 3623 | 916 | 301 | 221 | 417 |  |
|  | % of uniq | 100.00 | 16.19 | 0.65 | 0.17 | 1.14 | 0.00 | 81.94 | 0.12 | 0.03 | 0.01 | 0.01 | 0.01 |  |
| LH_FB2 | Total | 16214179 | 2761885 | 37280 | 412107 | 2240251 | 3219 | 11076942 | 336314 | 50059 | 4226 | 3917 | 17591 |  |
|  | % of Total | 100.00 | 17.03 | 0.23 | 2.54 | 13.82 | 0.02 | 68.32 | 2.07 | 0.31 | 0.03 | 0.02 | 0.11 |  |
|  | uniq | 3471738 | 661071 | 19011 | 5729 | 40544 | 143 | 2748827 | 3916 | 860 | 263 | 262 | 428 |  |
|  | % of uniq | 100.00 | 19.04 | 0.55 | 0.17 | 1.17 | 0.00 | 79.18 | 0.11 | 0.02 | 0.01 | 0.01 | 0.01 |  |
| LH_FB3 | Total | 14767229 | 1707724 | 37159 | 337623 | 1891796 | 3385 | 11063778 | 288583 | 28219 | 3773 | 3378 | 13670 |  |
|  | % of Total | 100.00 | 11.56 | 0.25 | 2.29 | 12.81 | 0.02 | 74.92 | 1.95 | 0.19 | 0.03 | 0.02 | 0.09 |  |
|  | uniq | 3177382 | 503304 | 17889 | 4508 | 36930 | 152 | 2617327 | 2979 | 737 | 213 | 226 | 353 |  |
|  | % of uniq | 100.00 | 15.84 | 0.56 | 0.14 | 1.16 | 0.00 | 82.37 | 0.09 | 0.02 | 0.01 | 0.01 | 0.01 |  |
| LH_DE1 | Total | 16072675 | 9651513 | 19198 | 309169 | 1493545 | 3670 | 4822524 | 245382 | 43761 | 2461 | 2228 | 15337 |  |
|  | % of Total | 100.00 | 60.05 | 0.12 | 1.92 | 9.29 | 0.02 | 30.00 | 1.53 | 0.27 | 0.02 | 0.01 | 0.10 |  |
|  | uniq | 2247954 | 902043 | 8954 | 4993 | 23897 | 148 | 1311152 | 3549 | 739 | 156 | 153 | 396 |  |
|  | % of uniq | 100.00 | 40.13 | 0.40 | 0.22 | 1.06 | 0.01 | 58.33 | 0.16 | 0.03 | 0.01 | 0.01 | 0.02 |  |
| LH_DE2 | Total | 22457369 | 4351890 | 50520 | 637750 | 3408405 | 5513 | 14459081 | 497304 | 89284 | 5756 | 8750 | 36656 |  |
|  | % of Total | 100.00 | 19.38 | 0.22 | 2.84 | 15.18 | 0.02 | 64.38 | 2.21 | 0.40 | 0.03 | 0.04 | 0.16 |  |
|  | uniq | 4071088 | 717324 | 23337 | 8150 | 53989 | 205 | 3273008 | 5485 | 1294 | 315 | 379 | 677 |  |
|  | % of uniq | 100.00 | 17.62 | 0.57 | 0.20 | 1.33 | 0.01 | 80.40 | 0.13 | 0.03 | 0.01 | 0.01 | 0.02 |  |
| LH_DE3 | Total | 20994218 | 2729421 | 52769 | 727438 | 3887600 | 4461 | 14142687 | 616223 | 67464 | 5835 | 7135 | 30781 |  |
|  | % of Total | 100.00 | 13.00 | 0.25 | 3.46 | 18.52 | 0.02 | 67.36 | 2.94 | 0.32 | 0.03 | 0.03 | 0.15 |  |
|  | uniq | 3996542 | 583796 | 23594 | 8299 | 53768 | 185 | 3332151 | 5922 | 1147 | 280 | 347 | 603 |  |
|  | % of uniq | 100.00 | 14.61 | 0.59 | 0.21 | 1.35 | 0.00 | 83.38 | 0.15 | 0.03 | 0.01 | 0.01 | 0.02 |  |

Notes: 3ADT&length filter: reads removed due to 3ADT not found and length with <18 nt and >25 nt were removed (for plants); length with<18 and >26 were remove (for animals). Junk reads: Junk: >=2N, >=7A, >=8C, >=6G, >=7T, >=10Dimer, >=6Trimer, or >=5Tetramer. Rfam: Collection of many common non-coding RNA families except micro RNA; <http://rfam.janelia.org>. Repeats: Prototypic sequences representing repetitive DNA from different eukaryotic species; <http://www.girinst.org/repbase>. Valid reads may not be equal to raw reads-3ADT&length filter-Junk reads ï¿½C mRNA ï¿½C Rfam-Repeats, because there are overlapped sequences between mRNAï¿½ï¿½Rfam and Repeats, details please refer to _comp_others.txt in fold 2_MappedData.
