# Supplementary material for: Integrated analysis of miRNAome transcriptome and degradome reveals miRNA-target modules governing floral florescence development and senescence across early- and late-flowering genotypes in tree peony
Source: Front Plant Sci. 2022 Dec 14;13:1082415. doi: 10.3389/fpls.2022.1082415 (PMC9795019; doi:10.3389/fpls.2022.1082415)
Supplement: Supplementary Figure 1 — Expressed miRNA detected across flower developmental stages and varieties in tree peony. (A) The distribution of expressed miRNAs across the four flower developmental stages (BS, IF, FB, DE) in FD. (B) The distribution of expressed miRNAs across the four flower developmental stages (BS, IF, FB, DE) in MU. (C) The distribution of expressed miRNAs across the four flower developmental stages (BS, IF, FB, DE) in LH. (D) Intersection of expressed miRNAs across flower developmental stages (BS, IF, FB, DE) and tree peony varieties (LH, MU and LH). (E) The distribution of expressed miRNAs across varieties (FD, MU and LH) at flower developmental stage BS. (F) The distribution of expressed miRNAs across varieties (FD, MU and LH) at flower developmental stage IF. (G) The distribution of expressed miRNAs across varieties (FD, MU and LH) at flower developmental stage FB. (H) The distribution of expressed miRNAs across varieties (FD, MU and LH) at flower developmental stage DE. (I) Intersection of expressed miRNAs across tree peony varieties (FD, MU and LH) and flower developmental stages (BS, IF, FB, DE). [file DataSheet_1.zip › Supplymentary files/Supplementary tables/Table S5 Summary of known and predicted miRNAs revealed in this study.docx]

Table S5 Summary of known and predicted miRNAs revealed in this study

| Library | Groups | gp1 | gp2a | gp2b | gp3 | gp4 |
| --- | --- | --- | --- | --- | --- | --- |
| Total | Pre-miRNA | 10 | 75 | 611 | 39 | 1709 |
|  | Unique miRNA | 15 | 106 | 632 | 43 | 1812 |
| FD_BS1 | Pre-miRNA | 10 | 52 | 219 | 18 | 688 |
|  | Unique miRNA | 14 | 71 | 226 | 17 | 715 |
| FD_BS2 | Pre-miRNA | 10 | 46 | 153 | 12 | 442 |
|  | Unique miRNA | 14 | 59 | 160 | 12 | 459 |
| FD_BS3 | Pre-miRNA | 10 | 52 | 220 | 15 | 526 |
|  | Unique miRNA | 15 | 68 | 226 | 14 | 547 |
| FD_DE1 | Pre-miRNA | 10 | 46 | 298 | 15 | 368 |
|  | Unique miRNA | 15 | 62 | 307 | 15 | 386 |
| FD_DE2 | Pre-miRNA | 10 | 46 | 264 | 11 | 311 |
|  | Unique miRNA | 15 | 60 | 270 | 11 | 326 |
| FD_DE3 | Pre-miRNA | 10 | 42 | 222 | 10 | 203 |
|  | Unique miRNA | 15 | 56 | 228 | 9 | 215 |
| FD_FB1 | Pre-miRNA | 10 | 43 | 228 | 12 | 284 |
|  | Unique miRNA | 15 | 59 | 232 | 11 | 298 |
| FD_FB2 | Pre-miRNA | 10 | 44 | 209 | 13 | 430 |
|  | Unique miRNA | 15 | 61 | 216 | 12 | 447 |
| FD_FB3 | Pre-miRNA | 10 | 48 | 213 | 13 | 443 |
|  | Unique miRNA | 15 | 63 | 220 | 12 | 462 |
| FD_IF1 | Pre-miRNA | 10 | 56 | 227 | 19 | 739 |
|  | Unique miRNA | 15 | 78 | 236 | 18 | 764 |
| FD_IF2 | Pre-miRNA | 10 | 50 | 196 | 11 | 428 |
|  | Unique miRNA | 15 | 67 | 202 | 10 | 446 |
| FD_IF3 | Pre-miRNA | 10 | 51 | 242 | 14 | 359 |
|  | Unique miRNA | 15 | 69 | 249 | 13 | 375 |
| LH_BS1 | Pre-miRNA | 10 | 46 | 190 | 14 | 370 |
|  | Unique miRNA | 15 | 63 | 198 | 14 | 387 |
| LH_BS2 | Pre-miRNA | 10 | 52 | 214 | 20 | 485 |
|  | Unique miRNA | 15 | 72 | 221 | 21 | 505 |
| LH_BS3 | Pre-miRNA | 10 | 50 | 193 | 14 | 623 |
|  | Unique miRNA | 14 | 68 | 198 | 13 | 645 |
| LH_DE1 | Pre-miRNA | 10 | 45 | 175 | 9 | 167 |
|  | Unique miRNA | 13 | 58 | 177 | 9 | 177 |
| LH_DE2 | Pre-miRNA | 10 | 54 | 249 | 16 | 555 |
|  | Unique miRNA | 14 | 72 | 254 | 15 | 577 |
| LH_DE3 | Pre-miRNA | 10 | 48 | 253 | 17 | 518 |
|  | Unique miRNA | 15 | 65 | 258 | 18 | 537 |
| LH_FB1 | Pre-miRNA | 10 | 47 | 196 | 12 | 264 |
|  | Unique miRNA | 13 | 64 | 204 | 13 | 279 |
| LH_FB2 | Pre-miRNA | 10 | 48 | 201 | 9 | 391 |
|  | Unique miRNA | 15 | 64 | 204 | 9 | 409 |
| LH_FB3 | Pre-miRNA | 10 | 47 | 198 | 15 | 338 |
|  | Unique miRNA | 13 | 63 | 201 | 14 | 353 |
| LH_IF1 | Pre-miRNA | 10 | 46 | 219 | 14 | 560 |
|  | Unique miRNA | 13 | 61 | 226 | 14 | 580 |
| LH_IF2 | Pre-miRNA | 10 | 43 | 227 | 15 | 518 |
|  | Unique miRNA | 15 | 60 | 233 | 15 | 537 |
| LH_IF3 | Pre-miRNA | 10 | 50 | 199 | 12 | 511 |
|  | Unique miRNA | 13 | 70 | 204 | 11 | 528 |
| MU_BS1 | Pre-miRNA | 10 | 54 | 233 | 18 | 616 |
|  | Unique miRNA | 15 | 74 | 241 | 18 | 643 |
| MU_BS2 | Pre-miRNA | 10 | 52 | 228 | 17 | 699 |
|  | Unique miRNA | 15 | 74 | 236 | 17 | 731 |
| MU_BS3 | Pre-miRNA | 10 | 54 | 240 | 16 | 837 |
|  | Unique miRNA | 15 | 73 | 249 | 16 | 873 |
| MU_DE1 | Pre-miRNA | 10 | 45 | 248 | 17 | 324 |
|  | Unique miRNA | 14 | 60 | 254 | 17 | 340 |
| MU_DE2 | Pre-miRNA | 10 | 42 | 298 | 14 | 302 |
|  | Unique miRNA | 15 | 56 | 305 | 14 | 315 |
| MU_DE3 | Pre-miRNA | 10 | 40 | 267 | 14 | 215 |
|  | Unique miRNA | 15 | 55 | 273 | 14 | 228 |
| MU_FB1 | Pre-miRNA | 10 | 51 | 219 | 16 | 663 |
|  | Unique miRNA | 15 | 66 | 226 | 16 | 691 |
| MU_FB2 | Pre-miRNA | 10 | 57 | 205 | 17 | 718 |
|  | Unique miRNA | 15 | 75 | 211 | 16 | 748 |
| MU_FB3 | Pre-miRNA | 10 | 54 | 258 | 16 | 793 |
|  | Unique miRNA | 15 | 75 | 266 | 15 | 824 |
| MU_IF1 | Pre-miRNA | 10 | 56 | 212 | 16 | 470 |
|  | Unique miRNA | 15 | 75 | 219 | 16 | 496 |
| MU_IF2 | Pre-miRNA | 10 | 48 | 216 | 16 | 437 |
|  | Unique miRNA | 15 | 65 | 225 | 16 | 459 |
| MU_IF3 | Pre-miRNA | 10 | 43 | 181 | 14 | 458 |
|  | Unique miRNA | 14 | 57 | 187 | 14 | 480 |

gp1: Reads map to specific miRNAs/pre-miRNAs in miRbase and the pre-miRNAs further map to the genome & EST.

gp2a: Reads map to selected miRNAs/pre-miRNAs in miRbase. The mapped pre-miRNAs do not map to the genome, but the reads (and of course the miRNAs of the pre-miRNAs) map to genome. The extended genome sequences from the genome loci may form hairpins.

gp2b: Reads were mapped to miRNAs/pre-miRNAs of selected species in miRbase and the mapped pre-miRNAs were not further mapped to genome, but the reads (and of course the miRNAs of the pre-miRNAs) were mapped to genome. The extended genome sequences from the genome loci may not form hairpins.

gp3: Reads map to slected miRNAs/pre-miRNAs in miRbase. The mapped pre-miRNAs do not map to the genome, and the reads do not map to the genome.

gp4: Reads do not map to selected pre-miRNAs in miRbase. But the reads map to genome & the extended genome sequences from genome may form hairpins.
