# Supplementary material for: Integrated analysis of miRNAome transcriptome and degradome reveals miRNA-target modules governing floral florescence development and senescence across early- and late-flowering genotypes in tree peony
Source: Front Plant Sci. 2022 Dec 14;13:1082415. doi: 10.3389/fpls.2022.1082415 (PMC9795019; doi:10.3389/fpls.2022.1082415)
Supplement: Supplementary Figure 1 — Expressed miRNA detected across flower developmental stages and varieties in tree peony. (A) The distribution of expressed miRNAs across the four flower developmental stages (BS, IF, FB, DE) in FD. (B) The distribution of expressed miRNAs across the four flower developmental stages (BS, IF, FB, DE) in MU. (C) The distribution of expressed miRNAs across the four flower developmental stages (BS, IF, FB, DE) in LH. (D) Intersection of expressed miRNAs across flower developmental stages (BS, IF, FB, DE) and tree peony varieties (LH, MU and LH). (E) The distribution of expressed miRNAs across varieties (FD, MU and LH) at flower developmental stage BS. (F) The distribution of expressed miRNAs across varieties (FD, MU and LH) at flower developmental stage IF. (G) The distribution of expressed miRNAs across varieties (FD, MU and LH) at flower developmental stage FB. (H) The distribution of expressed miRNAs across varieties (FD, MU and LH) at flower developmental stage DE. (I) Intersection of expressed miRNAs across tree peony varieties (FD, MU and LH) and flower developmental stages (BS, IF, FB, DE). [file DataSheet_1.zip › Supplymentary files/Supplementary tables/Table S9 Overview of degradome data revealed in this study.docx]

Table S9 Overview of degradome data revealed in this study

| Sample | FD (number) | FD (ratio) | LH (number) | LH (ratio) | MU (number) | MU(ratio) |
| --- | --- | --- | --- | --- | --- | --- |
| Raw Reads | 34133083 | / | 30564506 | / | 44319581 | / |
| Unique Raw Reads | 10248933 | / | 9044390 | / | 12359439 | / |
| reads < 15nt after removing 3 adaptor | 248517 | 0.73% | 209700 | 0.69% | 328652 | 0.74% |
| Mappable Reads | 33884566 | 99.27% | 30354806 | 99.31% | 43990929 | 99.26% |
| Unique reads < 15nt after removing 3 adaptor | 81006 | 0.79% | 72298 | 0.80% | 100007 | 0.81% |
| Unique Mappable Reads | 10167927 | 99.21% | 8972092 | 99.20% | 12259432 | 99.19% |
| Mapped Reads | 11993061 | 35.14% | 11051793 | 36.16% | 15761428 | 35.56% |
| Unique Mapped Reads | 2839809 | 27.71% | 2422302 | 26.78% | 3446321 | 27.88% |
| Number of input Transcript | 35687 | / | 35687 | / | 35687 | / |
| Number of Coverd Transcript | 21170 | 59.32% | 20626 | 57.80% | 21594 | 60.51% |
